# Nanoscintillator Coating: A Key Parameter That Strongly Impacts Internalization, Biocompatibility, and Therapeutic Efficacy in Pancreatic Cancer Models

**DOI:** 10.1002/smsc.202400041

**Published:** 2024-03-28

**Authors:** Clémentine Aubrun Fulbert, Frédéric Chaput, Sarah Stelse‐Masson, Maxime Henry, Benoit Chovelon, Sylvain Bohic, Dennis Brueckner, Jan Garrevoet, Christine Moriscot, Benoit Gallet, Julien Vollaire, Olivier Nicoud, Frédéric Lerouge, Sandrine Denis‐Quanquin, Xavier Jaurand, Thibault Jacquet, Anthony Nomezine, Véronique Josserand, Jean‐Luc Coll, Jean‐Luc Ravanat, Hélène Elleaume, Anne‐Laure Bulin

**Affiliations:** ^1^ Université Grenoble Alpes, INSERM, UA 07 Synchrotron Radiation for Biomedicine Grenoble France; ^2^ Université Grenoble Alpes INSERM U 1209, CNRS UMR 5309, Team Cancer Targets and Experimental Therapeutics, Institute for Advanced Biosciences 38000 Grenoble France; ^3^ Université de Lyon Laboratoire de Chimie, École Normale Supérieure de Lyon, Université Claude Bernard Lyon 1, CNRS UMR 5182 46 Allée d’Italie F69364 Lyon France; ^4^ Université Grenoble Alpes Département de Pharmacochimie Moléculaire, CNRS, UMR 5063 F‐38041 Grenoble France; ^5^ Service de Biochimie SB2TE Institut de Biologie et Pathologie, CHU Grenoble Alpes 38041 Grenoble France; ^6^ ESRF – The European Synchrotron Radiation Facility 38043 Grenoble France; ^7^ Deutsches Elektronen‐Synchrotron DESY Notkestr. 85 22607 Hamburg Germany; ^8^ Université Grenoble Alpes EMBL, Integrated Structural Biology Grenoble (ISBG), UAR 3518, CNRS, CEA 71 avenue des Martyrs F‐38042 Grenoble France; ^9^ Université Grenoble Alpes Institut de Biologie Structurale (IBS), CEA, CNRS 38000 Grenoble France; ^10^ Université Lyon 1 Centre Technologique des Microstructures F‐69622 Villeurbanne France; ^11^ Université Grenoble Alpes, CEA, CNRS, Grenoble INP, SyMMES UMR 5819 F‐38000 Grenoble France

**Keywords:** biocompatibility, nanoparticle functionalization, nanoscintillators, pancreatic cancer, radiation dose‐enhancement, radiotherapy

## Abstract

Pancreatic cancer is associated with a poor prognosis despite multimodal treatments. To improve the efficacy of radiotherapy, the use of nanoscintillators is emerging. Made of high‐Z elements, they absorb X‐rays more efficiently than tissues and can locally enhance the radiation dose provided they have accumulated near tumor cells. This study focuses on the role of the coating, a key parameter that controls both in vitro and in vivo properties of nanoparticles, including their internalization, biocompatibility, and therapeutic efficacy. Polyethylene glycol and tripolyphosphate molecules are used to coat lanthanum fluoride nanoscintillators, and their properties are evaluated on pancreatic cancer models. The experiments demonstrate a higher internalization of the nanoparticles when coated with tripolyphosphate, in both 2D and 3D culture models, correlating with greater efficacy under X‐rays, which may be associated with higher radiation dose‐enhancement. The nanoparticles are also injected intravenously in healthy or tumor‐bearing mice in order to study their toxicity, pharmacokinetics, and biodistribution. Despite a strong liver and spleen accumulation, especially for the tripolyphosphate‐coated nanoparticles, no toxicity is observed for either coating. Because they show promising radiation dose‐enhancement in vitro in both culture models and a limited toxicity in vivo, polyethylene glycol‐coated nanoparticles are good candidates for biomedical applications.

## Graphical abstract

Nanoscintillators are innovative radiotherapeutics developed to improve radiotherapy through various contributions, including radiation dose‐enhancement. Herein, it is demonstrated that nanoscintillator coating drives not only biological properties, including internalization, which directly correlates with therapeutic efficacy, but also biocompatibility and biodistribution in pancreatic tumor models. Preclinical studies show that a compromise can be reached between in vitro efficacy and in vivo biocompatibility.

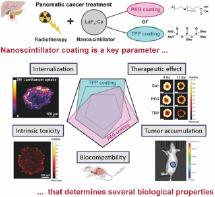

## 1 Introduction

Pancreatic ductal adenocarcinoma (PDAC) is the seventh leading cause of cancer death worldwide, with a 5 year survival rate below 10%.^[^ ^1^ ^]^ The steady rise in its incidence is partly attributed to an ageing population and lifestyle factors such as smoking, alcohol consumption, and obesity.^[^ ^2^ ^]^ Currently, the only curative treatment for PDAC is ablative surgery combined with adjuvant chemotherapy.^[^ ^3^ ^]^ However, many patients are diagnosed at an advanced stage with an unresectable tumor, and new treatments are urgently needed.

Radiotherapy (RT) has been studied as an adjuvant treatment for PDAC, but remains controversial and is currently only recommended for borderline cases of resectable PDAC.^[^ ^4^, ^5^ ^]^ While one American study reported a survival benefit when patients received chemoradiotherapy versus chemotherapy only,^[^ ^6^ ^]^ two European studies showed no benefit.^[^ ^7^, ^8^ ^]^ To improve the efficacy of RT for PDAC, one strategy is to specifically increase the dose delivered to the tumor. To this end, stereotactic body radiotherapy (SBRT) has shown promising results for PDAC, enabling good tumor control and increasing life expectancy compared to chemotherapy alone or to chemotherapy combined with conventional RT.^[^ ^5^, ^9^, ^10^ ^]^ However, as SBRT occasionally induces high toxicity,^[^ ^11^ ^]^ other approaches are needed to improve the dose contrast between tumor and healthy tissue. One strategy is to accumulate high‐Z elements in the tumor before irradiation to induce the so‐called radiation dose‐enhancement (RDE) effect. RDE is a physical effect intrinsically due to the presence of high‐Z elements during X‐ray exposure. These elements absorb orthovoltage (<250 keV) X‐rays more efficiently than tissue through photoelectric interactions. When photoelectric interactions occur, secondary electrons, including photo‐ and Auger electrons, are produced. This additional production of secondary electrons due to the high atomic number elements is the source of the physical dose increase. The mean free path of the electrons varies from a few nanometers (Auger electrons) to a few tens of micrometers (photoelectrons) as they interact with tissue and lose their energy by locally generating additional reactive oxygen species (ROS). To characterize the physical increase in radiation dose, the dose‐enhancement factor (DEF) is often used. It represents the ratio between the dose in tissues loaded with high‐Z elements and the dose in tissues without high‐Z elements. For example, with a DEF of 1.5, a 2 Gy dose delivered in the presence of nanoparticles (NPs) would have a therapeutic effect similar to that of 3 Gy delivered without NPs. RDE was first observed in the 70s with iodine contrast agents^[^ ^12^, ^13^, ^14^ ^]^ and revived 20 years ago when high‐Z element NPs were synthesized. Several studies ranging from Monte Carlo simulations^[^ ^15^ ^]^ to in vitro and in vivo studies^[^ ^16^, ^17^, ^18^, ^19^, ^20^, ^21^, ^22^, ^23^ ^]^ have demonstrated that such NPs could sensitize pancreatic cancer cells to X‐rays when NPs made of thorium,^[^ ^15^ ^]^ titanium,^[^ ^16^, ^17^ ^]^ manganese,^[^ ^18^ ^]^ yttrium,^[^ ^19^ ^]^ cerium,^[^ ^20^ ^]^ gadolinium,^[^ ^21^ ^]^ and gold^[^ ^22^, ^23^ ^]^ were incubated with tumor cells. Various effects were reported to explain this efficacy, including increased production of DNA damage^[^ ^16^, ^18^, ^21^, ^22^ ^]^ and ROS^[^ ^16^, ^17^, ^19^, ^20^ ^]^ enhanced apoptosis,^[^ ^16^, ^17^, ^18^, ^19^, ^20^, ^23^ ^]^ reduced cell proliferation,^[^ ^17^, ^22^ ^]^ and reduced survival fraction.^[^ ^16^, ^17^, ^20^, ^21^ ^]^ In vivo studies also demonstrated increased animal survival^[^ ^17^, ^21^ ^]^ and decreased tumor volumes.^[^ ^16^, ^17^, ^18^, ^19^, ^20^, ^21^ ^]^ Despite these encouraging results, the limited efficacy of RT in PDAC is thought to stem mainly from acquired radioresistance, potentially due to hypoxia or alterations in the DNA damage response, DNA repair machinery, and cell cycle checkpoint controls.^[^ ^24^ ^]^ Therefore, increasing the radiation dose may ultimately be insufficient and inducing combination effects relying on distinct biological pathways may be necessary.

Nanoscintillators composed of high‐Z elements recently emerged as promising radiotherapeutics. These NPs downconvert X‐rays into UV/visible light^[^ ^25^ ^]^ and gained interest for biomedical applications when they were proposed to induce photodynamic therapy in deep tissue under X‐ray irradiation.^[^ ^26^ ^]^ Since then, nanoscintillators were mainly investigated to induce not only X‐ray photodynamic therapy,^[^ ^27^ ^]^ but also UV‐C‐induced specific DNA damage^[^ ^28^, ^29^ ^]^ and more recently RDE.^[^ ^30^ ^]^ As these three therapeutic effects are based on different mechanisms of action, nanoscintillators could help combat radioresistance by activating various potentially synergistic effects.

When designing nanotherapeutics, achieving site‐specific bioavailability is challenged by several biological barriers at the systemic, microenvironmental, and cellular level.^[^ ^31^ ^]^ The size, shape, charge, and coating of the nanotherapeutics have been identified to strongly impact their ability to successfully cross these barriers.^[^ ^32^ ^]^

More specifically, the coating has been shown to directly influence NPs stability and dispersibility in biological media, cytotoxicity,^[^ ^33^ ^]^ cellular uptake,^[^ ^34^ ^]^ as well as in vivo biocompatibility, blood pharmacokinetics, and biodistribution.^[^ ^35^, ^36^ ^]^ Like most nanotherapeutics, nanoscintillators are expected to accumulate in the tumor through enhanced permeability and retention (EPR) effect, which has been extensively described in the literature.^[^ ^37^, ^38^ ^]^ Recently, questions have been raised about the relevance of this effect, as it is unclear whether nanomedicines can promote tumor accumulation via the EPR effect compared to free drugs.^[^ ^39^ ^]^ However, EPR effect has been confirmed in mouse models as well as in humans to promote tumor accumulation in comparison with normal tissues,^[^ ^39^ ^]^ which is the interesting property for nanoscintillators accumulation.

This study aims to decipher to what extent the coating can influence the biological properties of nanoscintillators and their in vitro therapeutic efficacy. To this end, a comprehensive study was carried out with two organic coatings, namely, sodium tripolyphosphate (TPP) and polyethylene glycol (PEG) chains, which is the gold standard coating used to reduce opsonization and guarantee stealth effect to nanotherapeutics.^[^ ^40^ ^]^ Both coatings were used to functionalize cerium‐doped lanthanum fluoride (LaF_3_:Ce) nanoscintillators. LaF_3_:Ce has long been investigated as a model scintillating material.^[^ ^41^ ^]^ Approximately 10 years ago, nanoformulations of LaF_3_:Ce were developed^[^ ^42^ ^]^ and started gaining interest for biomedical applications because of their interesting scintillating properties.^[^ ^43^ ^]^ More recently, we demonstrated that small LaF_3_:Ce NPs, synthesized by a solvothermal route, induced a potent RDE in glioblastoma models.^[^ ^30^ ^]^ In this article, the effect of two different coatings on the therapeutic efficacy of these LaF_3_:Ce NPs was investigated. The impact of the coating was first assessed in vitro on cell internalization, toxicity, and therapeutic efficacy under X‐rays, and then in vivo on biocompatibility, blood pharmacokinetics, and biodistribution. In vitro experiments were carried out on human pancreatic cancer cell lines grown as adherent monolayers (2D) and spheroids (3D), while in vivo experiments were conducted on healthy and mice bearing orthotopic pancreatic tumors.

## 2 Results

### 2.1 Characterization of LaF_3_:Ce NPs That Exhibit a Pure Crystal Phase

**Figure 1:**
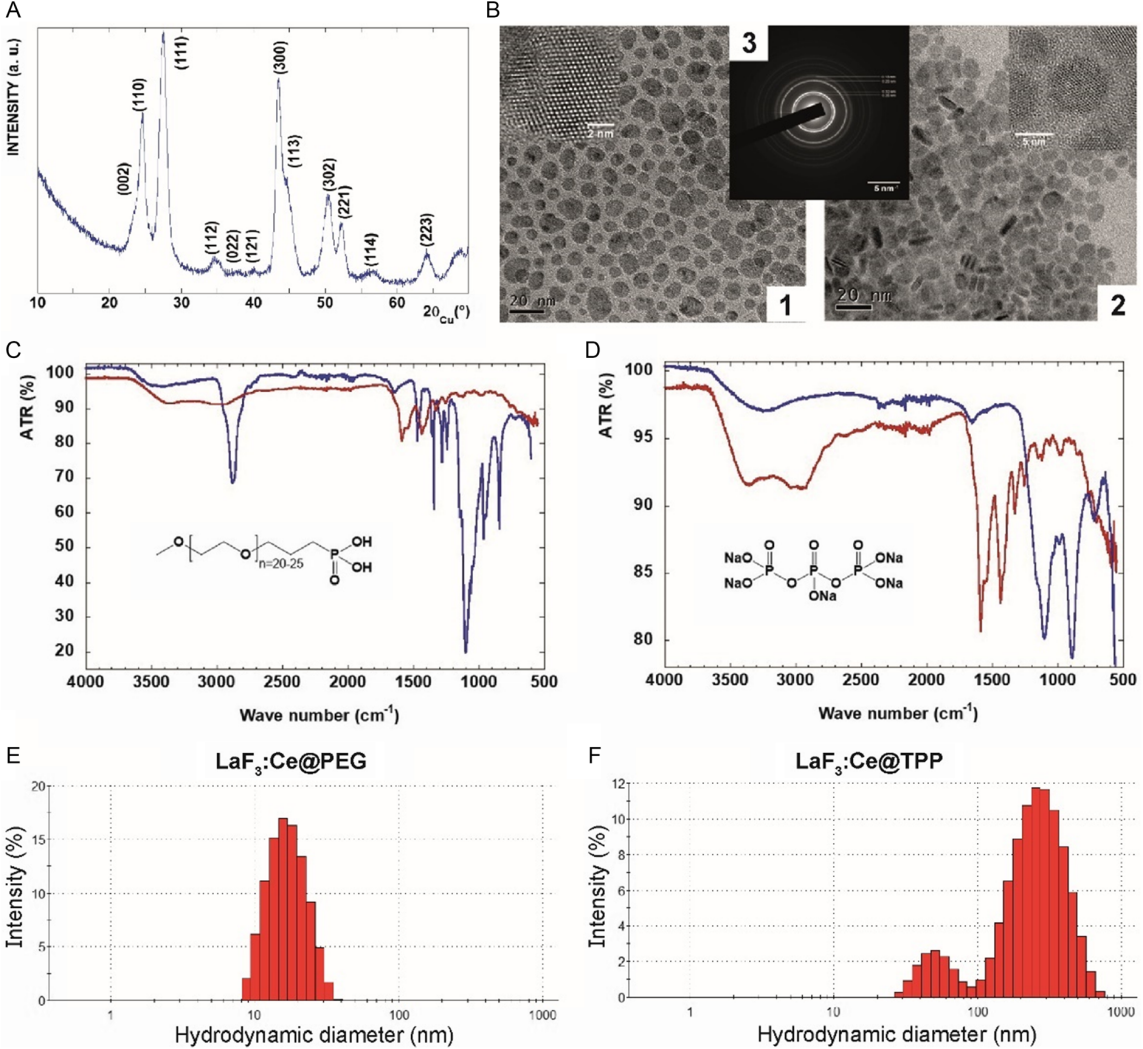
PEG‐ and TPP‐coated LaF_3_:Ce NPs were successfully synthesized. A) XRD pattern of LaF_3_:Ce nanopowder solvothermally obtained in 2‐pyrrolidinone at 170 °C for 1 h. B) TEM images of LaF_3_:Ce NPs after surface modification with PEG (1) or TPP (2) molecules, scale = 20 nm. Electronic diffraction of nanodiscs (3), scale = 5 nm. C) FTIR‐ATR spectra of the LaF_3_:Ce NPs before (red) and after (blue) PEGylation. D) FTIR‐ATR spectra of the LaF_3_:Ce NPs before (red) and after (blue) TPP grafting. E,F) Hydrodynamic diameters of LaF_3_:Ce NPs in PBS with PEG (E) and TPP (F) coating, measured by DLS.

The X‐ray diffraction (XRD) pattern of LaF_3_:Ce NPs isolated in powder form showed distinct diffraction peaks whose positions and intensities match the standard pattern of pure hexagonal LaF_3_ crystal (tysonite structure, space group $P\bar{3}c1$; ICDD card 01‐082‐0690) (**Figure** **1**A). No additional peak was observed, ruling out the existence of a secondary phase. The broadening of the diffraction peaks confirms the nanocrystalline nature of the powder.

After functionalization, transmission electron microscopy (TEM) images were acquired (Figure 1B) and highlighted the crystallinity and nanodisc morphology of LaF_3_:Ce NPs coated with PEG (Figure 1B1) or TPP (Figure 1B2) molecules. Statistical analysis of several hundreds of NPs provided a mean diameter of 10 nm and a standard deviation of 3.5 nm. Forty‐two discs were imaged at their edges to calculate the mean thickness, estimated at 3.8 nm with a standard deviation of 0.5 nm. High‐resolution TEM images showed a monocrystalline structure with no twin boundary (Figure 1B1,B2). Also, while PEG‐coated NPs appeared well‐individualized (Figure 1B1), TPP‐coated NPs tended to aggregate (Figure 1B2), which can be explained by the absence of steric repulsion when using TPP molecules, compared to PEG chains. In addition, phosphorus atoms in TPP molecules can interact wholly or partially with cations present on the surface of LaF_3_:Ce NPs, thus providing a surface charge to the NPs. Finally, the electron diffraction pattern showed no other phases or impurities (Figure 1B3), consistently with the XRD results.

The NPs composition evaluated by energy‐dispersive spectrometry (EDS) was in accordance with the targeted composition (La_0.9_Ce_0.1_F_3_). It was confirmed by inductively coupled plasma mass spectrometry (ICP‐MS) analysis that provided a lanthanum/cerium ratio of 8.73 (±0.01, SEM) for PEG‐coated NPs and 8.91 (±0.04, SEM) for TPP‐coated NPs.

Signals between 1350 and 1750 cm^−1^ and between 2650 and 3700 cm^−1^ on the spectra measured by attenuated total reflection Fourier transform infrared (ATR‐FTIR) spectroscopy (Figure 1C,D), demonstrated that small organic capping molecules coming from the solvent (2‐pyrrolidinone or its open form γ‐aminobutyric acid) were present on the surface of the NPs. ATR‐FTIR spectroscopy was then used to demonstrate the effective grafting of PEG (Figure 1C) or TPP (Figure 1D) groups on the surface of the NPs. After PEGylation, the infrared spectrum (Figure 1C, blue) showed typical signatures of PEG species, with bands at 2880 cm^−1^ and around 1100 cm^−1^ for ether, C—H and C—O—C stretching modes, respectively. The bands assigned to solvent molecules were no longer present. After TPP grafting, the infrared spectrum (Figure 1D, blue) showed the infrared absorption bands of the triphosphoric acid salt, replacing the bands of the solvent molecules. The FTIR spectrum of pure sodium TPP showed characteristic bands at 1218 cm^−1^ (stretching vibration of P=O), 1136 cm^−1^ (symmetric and anti‐symmetric stretching vibration of O—P=O), 1091 cm^−1^ (symmetric and asymmetric stretching vibration of the PO_3_), and 886 cm^−1^ (stretching vibration of P—O—P bridge).^[^ ^44^ ^]^ The amount of ligand on the NP surface was assessed by thermogravimetric analysis. The sample of PEGylated NPs showed a significant mass loss (≈30%) in the 150–400 °C range, attributed to the decomposition of the organic part. On the contrary and as expected, a small mass loss (<4%) was observed for the NPs with the inorganic TPP ligand. The coated NPs were also analyzed by nuclear magnetic resonance (NMR), in solution in deuterated water. The ^31^P spectrum in solution confirmed the presence of PEG and TPP at the surface of the NPs (Figure S1 and S2, Supporting Information).

The stability of the NPs was evaluated by dynamic light scattering (DLS) performed on LaF_3_:Ce NPs suspended in phosphate buffer saline (PBS), culture medium, and mouse plasma. In PBS, the hydrodynamic diameter of LaF_3_:Ce NPs was 22 nm with a polydispersity index (PDI) of 0.19 for PEG‐coated NPs (Figure 1E) and 180 nm with a PDI of 0.32 for the TPP‐coated NPs (Figure 1F). These results were consistent with the TEM images that showed well‐dispersed PEG‐coated NPs and aggregated TPP‐coated NPs. Unlike PEG‐coated NPs that were stable regardless of the medium (hydrodynamic diameter < 45 nm, PDI < 0.45), TPP‐coated NPs tended to aggregate in culture medium (hydrodynamic diameter = 790 nm, PDI = 0.55) and mouse plasma (hydrodynamic diameter = 257 nm, PDI = 0.90).

Zeta potentials (*ζ*) measured in PBS were −2.24 mV and −23.13 mV for PEG‐ and TPP‐coated NPs, respectively.

### 2.2 LaF_3_:Ce NPs Accumulate in Cytoplasmic Vesicles and TPP‐Coated NPs Are More Efficiently Internalized Compared to PEG‐Coated NPs

LaF_3_:Ce NPs are only fluorescent upon far‐UV or X‐ray excitation and cannot be imaged by classical confocal microscopy. To visualize their intracellular distribution, two complementary methods were used: X‐ray fluorescence (XRF) microscopy and TEM. Whereas XRF microscopy is quantitative (Figure S3, Supporting Information), it does not provide confocal resolution and therefore does not inform about the actual internalization of the NPs. On the contrary, TEM only provides a qualitative localization of the NPs visualized as dark spots.

**Figure 2:**
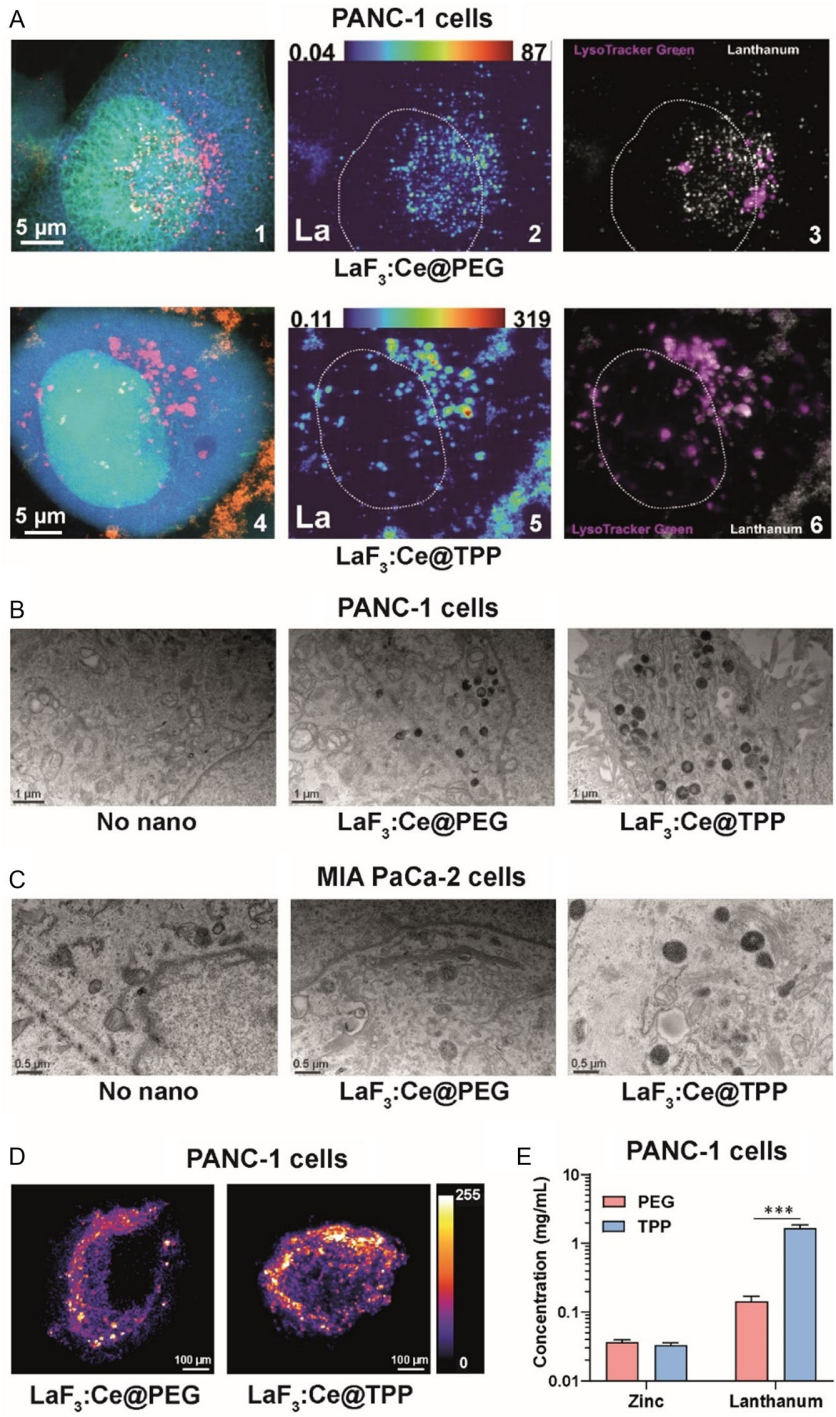
LaF_3_:Ce NPs internalize in lysosomes and diffuse throughout spheroids. A) XRF images of PANC‐1 cells previously incubated for 24 h with 0.1 mg mL^−1^ PEG‐ (1, 2, 3) or TPP‐ (4, 5, 6) coated NPs (scale = 5 μm): 1,4) zinc (nucleus, green), potassium (cytoplasm, blue), and lanthanum (NPs, red); 2,5) lanthanum; 3,6) lysosomes (lysotracker green, purple) and lanthanum (NPs, white). B,C) TEM images of PANC‐1 (B, scale = 1 μm) and MIA PaCa‐2 cells (C, scale = 0.5 μm) incubated for 24 h with PBS or 0.1 mg mL^−1^ NPs. D) CT images of XRF emission of lanthanum in PANC‐1 spheroids incubated for 24 h with 0.1 mg mL^−1^ NPs (scale = 100 μm). E) Zinc (cell) and lanthanum concentrations quantified using the XRF microtomography images.

XRF images depict zinc (nucleus, green), potassium (cytoplasm, blue), and lanthanum (LaF_3_:Ce NPs, red) (**Figure** **2**A1,A4) and show an accumulation of NPs as small and large clusters for PEG‐ and TPP‐coated NPs, respectively (Figure 2A2,A5). XRF data showed a stronger accumulation of lanthanum for TPP‐ than PEG‐coated NPs and a specific accumulation of the NPs in lysosomes, especially when coated with TPP (Figure 2A3, A6, and S4, Supporting Information). TEM images confirmed the accumulation of the NPs in cytoplasmic vesicles (Figure 2B,C) and a stronger internalization of TPP‐ compared to PEG‐coated NPs.

### 2.3 LaF_3_:Ce NPs Diffuse throughout the Spheroid and Accumulate Well within 3D Models

XRF microtomography performed on PANC‐1 spheroids provided qualitative and quantitative distribution of zinc and lanthanum. The computed tomography (CT) images showed that NPs were able to diffuse through the spheroids. Moreover, TPP‐coated NPs accumulated 11.5‐fold more than PEG‐coated NPs in the spheroids, with lanthanum concentrations of 1.6 and 0.14 mg cm^−3^, respectively.

### 2.4 LaF_3_:Ce NPs Show No Toxicity on Cells Grown as Monolayers When Incubated for 24 h at Concentrations up to 1 mg mL^−1^ in Culture Medium

MTS assays were performed on PANC‐1 (**Figure** **3**A) and MIA PaCa‐2 (Figure 3B) cells incubated 24 h with increasing concentrations of NPs. Cell viability stayed above 95% for concentrations of up to 1 mg mL^−1^. IC_50_ values measured on PANC‐1 cells reached 4.7 and 2.9 mg mL^−1^ for PEG‐ and TPP‐coated NPs, respectively, whereas on MIA PaCa‐2 cells, they reached 5.6 and 4.0 mg mL^−1^ for PEG‐ and TPP‐coated NPs, respectively.

Figure 3LaF_3_:Ce NPs induce a strong RDE effect in 2D cultures. A,B) Cell viability after incubation with increasing doses of NPs in PANC‐1 (A) and MIA PaCa‐2 cells (B) assessed by MTS. Nonlinear regression fits: [inhibitor] versus response model. C,D) Lanthanum concentration measured in each cell line by ICP‐MS after 24 h incubation with 1 mg mL^−1^ NPs (C) or 1 h incubation with 5 mg mL^−1^ NPs (D). E) Timeline of the clonogenic assay: cells were incubated with 5 mg mL^−1^ NPs for 1 h, irradiated with X‐ray, rinsed and seeded for colony formation. Created with BioRender.com. F–I) Representative images of PANC‐1 (F) and MIA PaCa‐2 (H) colonies stained with crystal violet (scale = 1 cm). Survival fraction calculated for PANC‐1 (G) and MIA PaCa‐2 (I) cells. Data were normalized on the 0 Gy condition and fitted with the linear quadratic cell death model. (*) indicates *p* < 0.05, (**) indicates *p* < 0.01, and (***) indicates *p* < 0.001.

**Figure:**
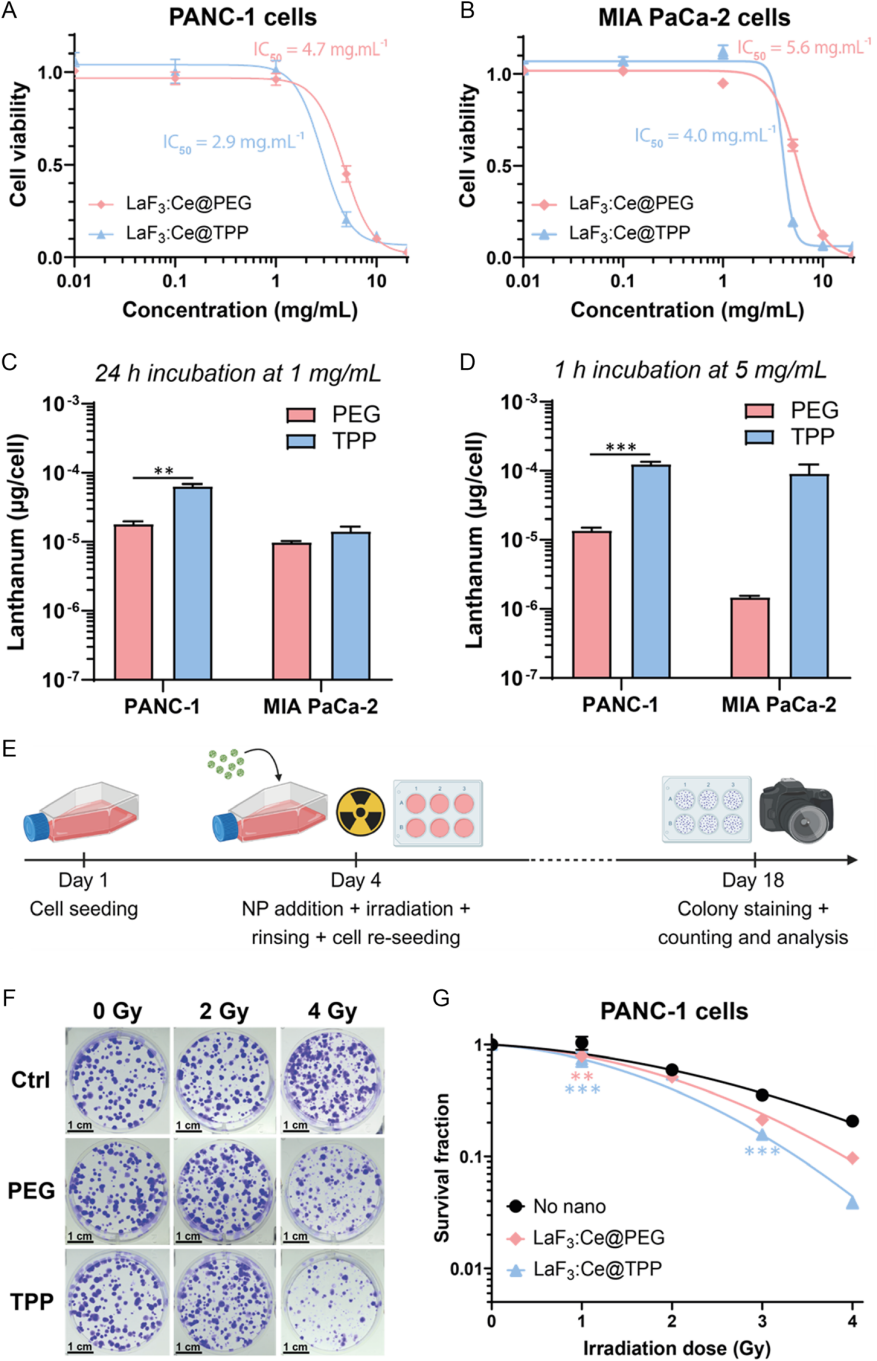

**Figure:**
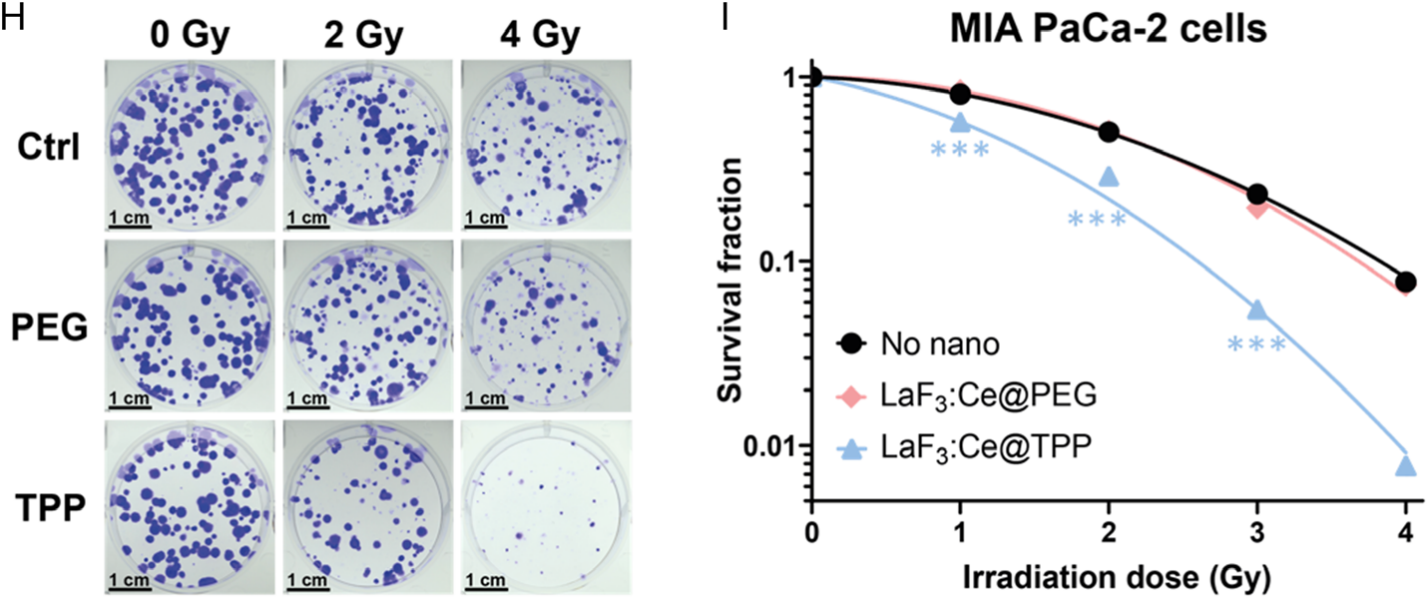

### 2.5 TPP‐Coated LaF_3_:Ce NPs Induce a Stronger RDE Effect in Monolayers than PEG‐Coated NPs

Because RDE effect strongly depends on the intracellular concentration in heavy elements,^[^ ^45^ ^]^ two incubation conditions were compared: 24 h at 1 mg mL^−1^ (Figure 3C) and 1 h at 5 mg mL^−1^ (Figure 3D). The lanthanum and cerium concentrations were measured by ICP‐MS. After 1 h incubation with 5 mg mL^−1^, intracellular accumulation of lanthanum was 9 and 62 times higher for TPP‐ versus PEG‐coated NPs for PANC‐1 and MIA PaCa‐2 cells, respectively (Table S1, Supporting Information, Figure 3D). After 24 h incubation with 1 mg mL^−1^, these ratios decreased to 3.5 and 1.4 for PANC‐1 and MIA PaCa‐2 cells, respectively (Figure 3C). Similar results were obtained for cerium (Figure S5, Supporting Information) and the ratios between lanthanum and cerium concentrations were close to the expected value of 9. The therapeutic efficacy of LaF_3_:Ce NPs was assessed by clonogenic assays after 1 h incubation at 5 mg mL^−1^ (Figure 3E). Whatever the coating, the NPs strongly enhanced the effect of X‐ray irradiation on PANC‐1 cells, whose ability to proliferate was severely impaired (Figure 3F,G). However, only the TPP‐coated NPs significantly decreased the proliferation of MIA PaCa‐2 cells after X‐ray irradiation (Figure 3H,I). Experimental DEFs were calculated using linear quadratic regression fits applied to the survival data, as detailed in S6, Supporting Information. The radiobiologic *α* and *β* parameters extracted from these fits are presented **Table** **1**.

**Table 1:** Linear quadratic parameters and DEFs extracted from clonogenic assays

	Control			LaF_3_:Ce@PEG				LaF_3_:Ce@TPP			
	*α* [Gy^−1^]	*β* [Gy^−2^]	*α*/*β* [Gy]	*α* [Gy^−1^]	*β* [Gy^−2^]	*α*/*β* [Gy]	DEF	*α* [Gy^−1^]	*β* [Gy^−2^]	*α*/*β* [Gy]	DEF
PANC‐1	0.12	0.07	1.62	0.10	0.13	0.80	1.24	0.14	0.16	0.85	1.45
MIA PaCa‐2	0.07	0.14	0.53	0.00	0.17	0.00	1.03	0.36	0.20	1.75	1.42

For PANC‐1, the *α*/*β* ratio decreased in presence of PEG‐ or TPP‐coated NPs compared to the controls. For MIA PaCa‐2, this ratio decreased to almost 0 with PEG‐coated NPs and strongly increased with TPP‐coated NPs. With PEG‐coated NPs, the RDE effect occurred only for PANC‐1, with a calculated DEF of 1.24 versus 1.03 for MIA PaCa‐2. In contrast, TPP‐coated NPs induced a strong RDE effect for both cell lines, with DEFs of 1.45 and 1.42 for PANC‐1 and MIA PaCa‐2, respectively.

### 2.6 Tumor Spheroids Can Be Safely Incubated for 24 h with 1 mg mL^−1^ NPs

A live/dead assay was used to evaluate the viability of tumor spheroids previously incubated for 24 h with increasing concentrations of LaF_3_:Ce NPs (**Figure** **4**A,B).^[^ ^30^, ^46^ ^]^ PEG‐coated NPs are well tolerated (viability >95%) up to 2.5 mg mL^−1^. TPP‐coated NPs started to affect the spheroid viability for concentrations above 1 mg mL^−1^. For NP concentrations ≥10 mg mL^−1^, the spheroids lost their integrity, making viability analysis impossible (data not shown). IC_50_ values were calculated for PEG‐ and TPP‐coated NPs and reached, respectively, 6.4 and 2.7 mg mL^−1^ for PANC‐1 spheroids, and 5.4 and 3.0 mg mL^−1^ for MIA PaCa‐2 spheroids.

Figure 4LaF_3_:Ce NPs induce a strong RDE effect in pancreatic tumor spheroids. A,B) Viability of PANC‐1 (A) and MIA PaCa‐2 (B) spheroids previously incubated with increasing concentrations of LaF_3_:Ce NPs, measured using a live/dead assay. Data were adjusted using a nonlinear [inhibitor] versus response model. C,D) Lanthanum and cerium concentrations measured by ICP‐MS in PANC‐1 (C) and MIA PaCa‐2 (D) spheroids. E) Timeline used to assess the therapeutic effect of NPs under X‐ray in tumor spheroids: after 24 h incubation with 1 mg mL^−1^ NPs, spheroids were irradiated, rinsed and maintained for 5 days before undergoing a live/dead assay. Created with BioRender.com. F,H) Representative viability heatmaps of PANC‐1 (F) and MIA PaCa‐2 (H) spheroids irradiated at 0, 8 and 12 Gy (scale = 250 μm). G,I) Spheroid viability of PANC‐1 (G) and MIA PaCa‐2 (I) spheroids. Data were normalized on the 0 Gy condition; linear regression fits were applied. (*) indicates *p* < 0.05, (**) indicates *p* < 0.01, and (***) indicates *p* < 0.001.

**Figure:**
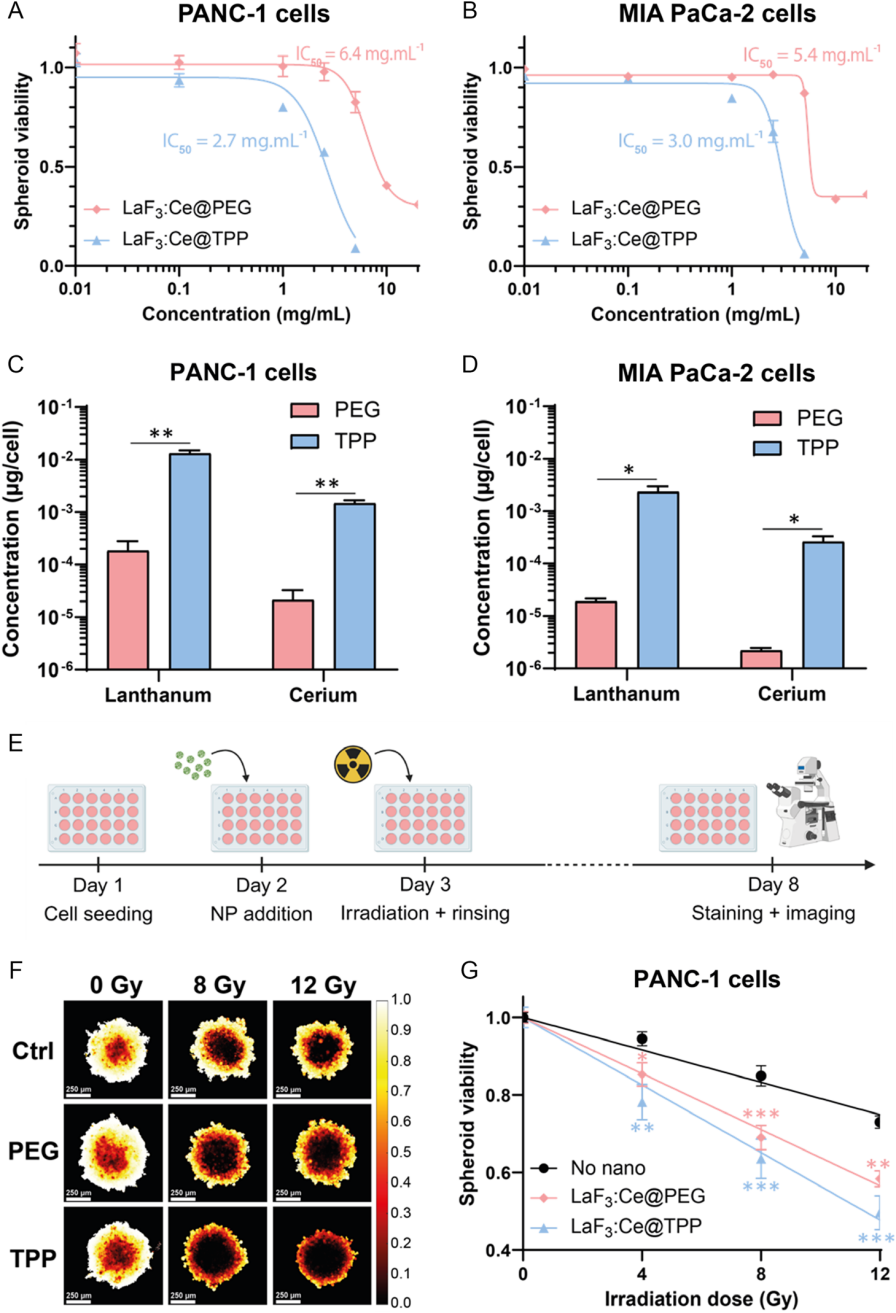

**Figure:**
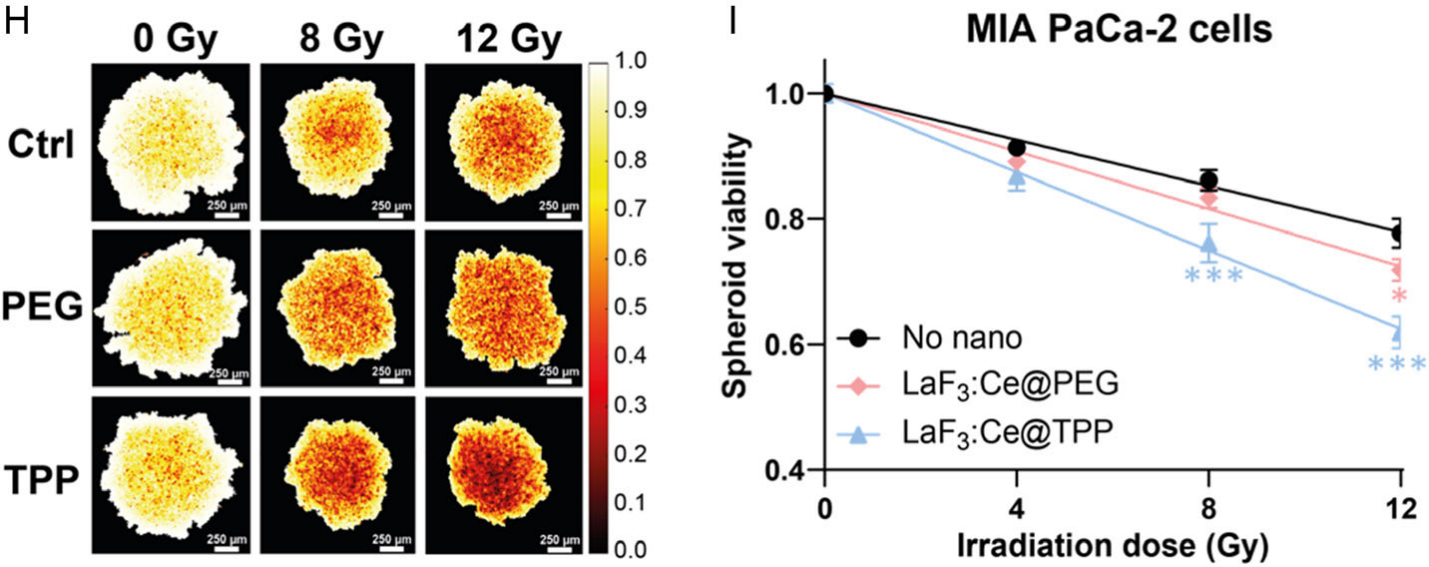

### 2.7 TPP‐Coated NPs Accumulate More Efficiently in Tumor Spheroids Than PEG‐Coated NPs

After 24 h incubation with 1 mg mL^−1^ NPs, the concentration of lanthanum accumulated in spheroids was assessed by ICP‐MS (Figure 4C,D and Table S1, Supporting Information). TPP‐coated NPs accumulated significantly more compared to PEG‐coated NPs: 71 times more in PANC‐1 spheroids (Figure 4C) and 122 times more in MIA PaCa‐2 spheroids (Figure 4D). However, the concentrations measured for TPP‐coated NPs are probably overestimated due to experimental difficulties of collecting the spheroids without simultaneously harvesting NPs aggregates, which remained in suspension despite the washing steps. For each condition, the lanthanum/cerium ratio was between 8.6 and 8.9, in agreement with the expected value of 9.

### 2.8 LaF_3_:Ce NPs Induce a Significant RDE Effect in Tumor Spheroids

To assess the RDE effect induced by LaF_3_:Ce NPs in spheroids, live/dead assays were performed (Figure 4E). After 24 hours incubation with 1 mg mL^−1^ NPs, spheroids were exposed to increasing doses of X‐rays (0–12 Gy). LaF_3_:Ce NPs significantly reduced spheroid viability with both coatings in PANC‐1 spheroids (Figure 4F,G) and with TPP‐coating only in MIA PaCa‐2 spheroids (Figure 4H,I). A significant, yet milder effect, was reported for PEG‐coated NPs in MIA PaCa‐2 spheroids (Figure 4H,I). PEG‐ and TPP‐coated NPs also increased spheroid necrosis upon X‐ray irradiation for both cell lines (Figure S6, Supporting Information), whereas spheroid size was only reduced by PEG‐coated NPs for PANC‐1 spheroids (Figure S7, Supporting Information). DEFs were calculated using the slope (*γ* parameter) extracted from the linear regression models applied to the spheroid viability data; DEF and *γ* parameters are provided in **Table** **2**.

**Table 2:** Simple linear parameters and DEFs

	Control	LaF_3_:Ce@PEG		LaF_3_:Ce@TPP	
	*γ* [Gy^−1^]	*γ* [Gy^−1^]	DEF	*γ* [Gy^−1^]	DEF
PANC‐1	0.021	0.036	1.73	0.044	2.08
MIA PaCa‐2	0.018	0.023	1.25	0.031	1.70

Both PEG‐ and TPP‐coated NPs induced a strong RDE, with DEFs of 1.73 and 2.08 for PANC‐1 spheroids, and 1.25 and 1.70 for MIA PaCa‐2 spheroids, respectively. For both cell lines, the experimental DEFs were higher with TPP‐coated NPs compared to PEG‐coated ones.

### 2.9 DEF Correlates with Lanthanum Concentration in 2D and 3D Models

**Figure 5:**
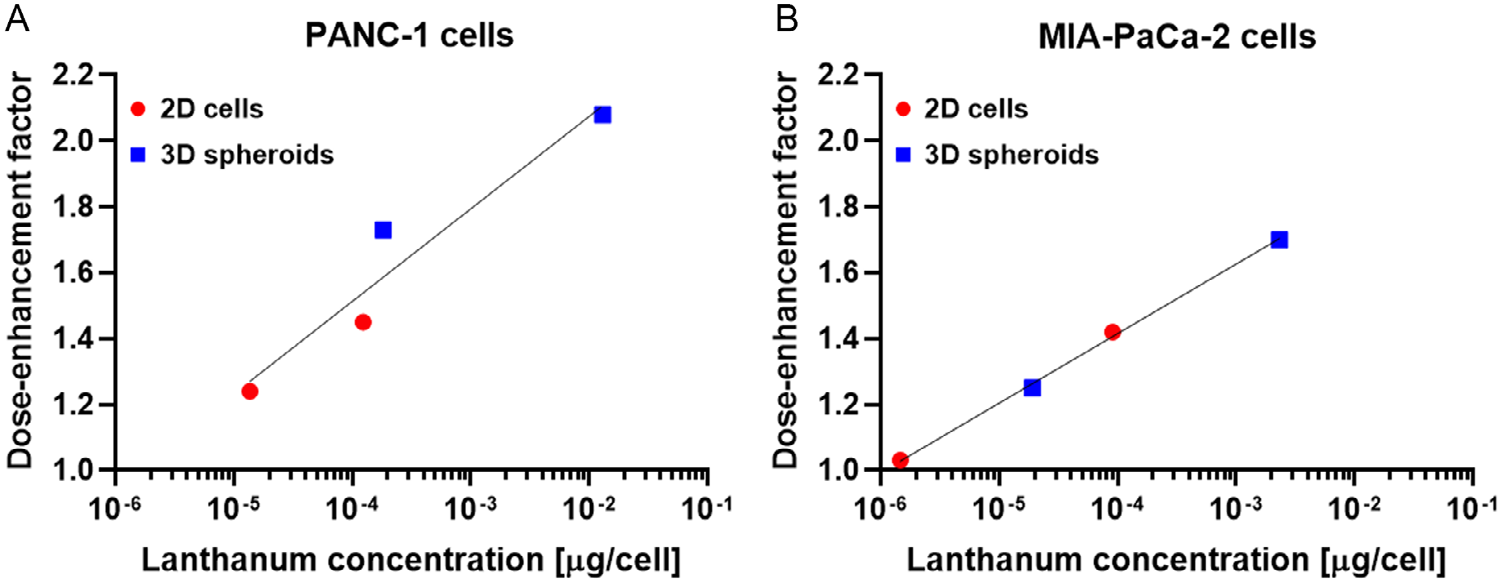
Experimental DEF plotted as a function of the lanthanum concentration measured by ICP‐MS in 2D (red dots) and 3D (blue squares) models. Results are shown for A) PANC‐1 and B) MIA PaCa‐2 cells.

The experimental DEFs measured from the clonogenic or the spheroids assays were plotted as a function of the intracellular lanthanum concentration measured by ICP‐MS (**Figure** **5**). A semilog function fitted the data with a *R* ^2^ coefficient of 0.92 and 0.99 for the PANC‐1 and MIA PaCa‐2 cells, respectively, demonstrating a strong correlation between the intracellular, or intraspheroid lanthanum concentration and the DEF.

### 2.10 LaF_3_:Ce NPs Are Safe to Inject Intravenously in Healthy Mice up to 200 mg kg^−1^

Intravenous (iv) injection of 200 mg kg^−1^ PEG‐ or TPP‐coated NPs induced no alteration in mice body weight (Figure S8, Supporting Information), no signs of pain, nor changes in behavior or survival.

#### 2.10.1 LaF_*3*_ *:Ce NPs Tend to Accumulate in the Liver and Spleen of Healthy Mice*

**Figure 6:**
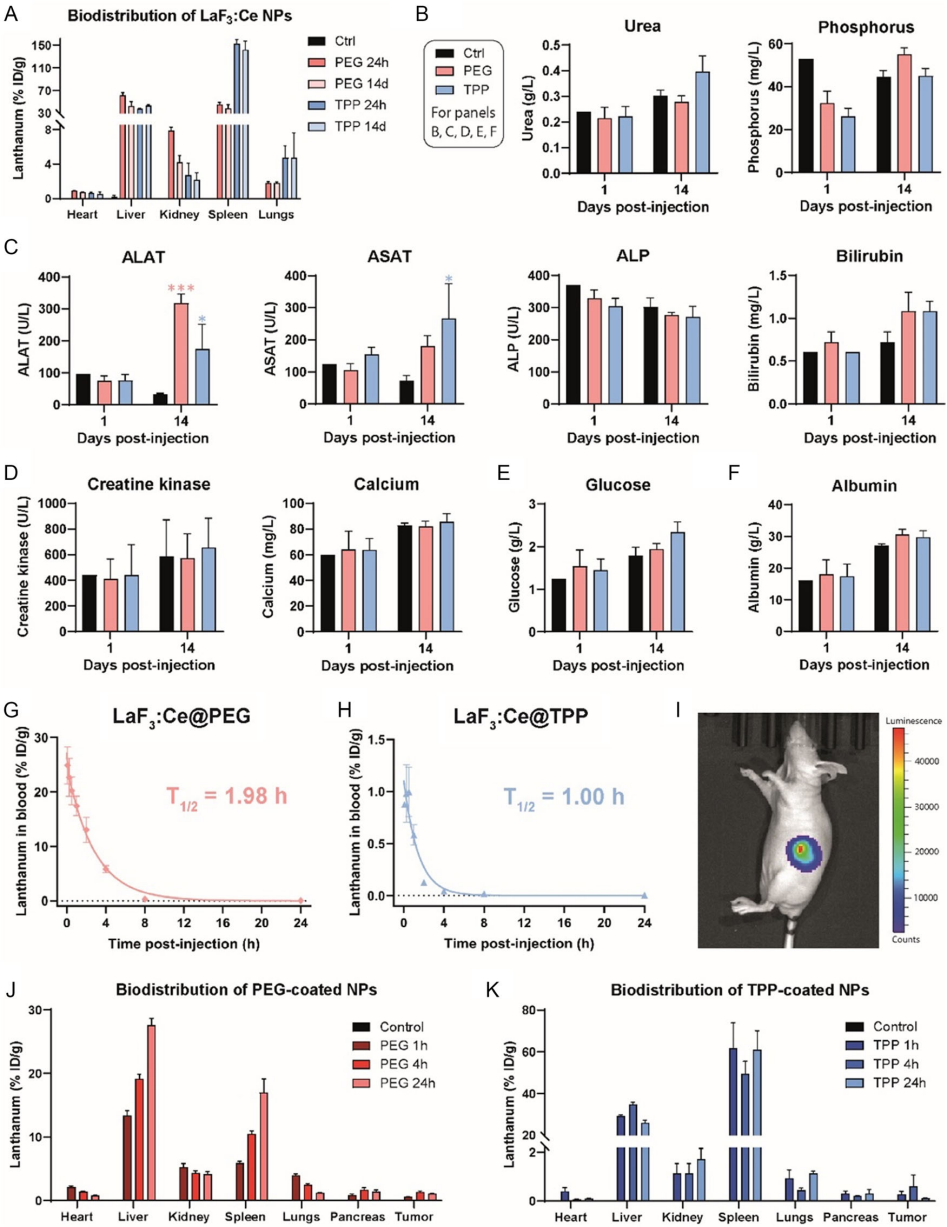
LaF_3_:Ce NPs can safely be intravenously injected in mice at 200 mg kg^−1^. A) Biodistribution of PEG‐ and TPP‐coated NPs after intravenous injection in BALB/c mice, *N* = 3 mice/group. Lanthanum concentration was measured by ICP‐MS in organs collected 24 h and 14 days after injection, and normalized as %ID g^−1^ in tissue. B–F) Plasma biochemistry on blood samples collected 24 h and 14 days postinjection. Analyses were related to renal (B), hepatic (C), and muscular functions (D), and metabolism (E) and nutritional status (F), *N* = 5 mice/group. ALAT, alanine transaminase; ASAT, aspartate transaminase; ALP, alkaline phosphatase. G,H) Pharmacokinetic profile of PEG‐ (G) and TPP‐coated (H) NPs, *N* = 3 mice/group. Lanthanum concentration in blood was measured by ICP‐MS and normalized as %ID g^−1^ in blood. A two‐phase decay model was used to fit the data. I) Representative bioluminescence image of a tumor‐bearing mouse acquired 34 days after orthotopic implantation of Luc‐PANC‐1 cells. J,K) Biodistribution of PEG‐ (J) and TPP‐coated NPs (K) after injection in tumor‐bearing mice, *N* = 5 mice/group. Tissues were collected 1, 4, and 24 h after injection and analyzed by ICP‐MS.

NPs mainly accumulated in the liver and spleen, as measured by ICP‐MS (**Figure** **6**A). Twenty‐four hours after injection, 62%ID g^−1^ (percent injected dose per gram of tissue) and 37%ID g^−1^ lanthanum were measured in the liver for PEG‐ and TPP‐coated NPs, respectively. These values reached 42%ID g^−1^ 14 days postinjection for both coatings. In the spleen, the coating strongly influenced the uptake as 45% and 152%ID g^−1^ lanthanum were measured 24 h postinjection for PEG‐ and TPP‐coated NPs, respectively. These values only slightly decreased over 14 days. Lanthanum was also found in the kidney with a higher concentration for PEG‐coated NPs and in the lungs with a higher concentration for TPP‐coated. Over time, lanthanum concentration decreased in the kidney, yet remained stable in the lungs.

#### 2.10.2 Injection of 200 mg kg^*−1*^ *LaF*_*3*_ *:Ce NPs Did Not Severely Impair Plasma Biochemistry*

Biochemistry analysis was performed on plasma collected 24 h and 14 days after the injection of 200 mg kg^−1^ LaF_3_:Ce NPs (Figure 6B–F). After 24 h, no significant effect was reported on any of the measured parameters. After 14 days, ALAT and ASAT levels were significantly increased by PEG‐ or TPP‐coated NPs and by TPP‐coated NPs, respectively (Figure 6C). No significant change was observed in any other functions.

### 2.11 PEG‐Coated NPs Circulate Twice as Long as TPP‐Coated NPs in the Blood

The pharmacokinetic profile of LaF_3_:Ce NPs was evaluated by ICP‐MS. It showed progressive elimination from the bloodstream over time, for both PEG‐ (Figure 6G) and TPP‐coated NPs (Figure 6H). The elimination half‐life in blood was 2 and 1 h for PEG‐ and TPP‐coated NPs, respectively. Not only did PEG‐coated NPs circulate longer than TPP‐coated NPs, but the concentration of lanthanum in blood 5 min after injection was also much higher, with 25%ID g^−1^ versus less than 1%ID g^−1^.

### 2.12 PEG‐Coated NPs Accumulate More Efficiently in Orthotopic Tumors Than TPP‐Coated NPs

The biodistribution of LaF_3_:Ce NPs intravenously injected was investigated in an orthotopic model of pancreatic cancer (Luc‐PANC‐1 cells) implanted in nude mice. Tumor implantation did not alter mice body weight (Figure S9, Supporting Information) nor induced signs of pain, changes in behavior or in survival. NPs were injected 35 days postimplantation, after the randomization of animals based on tumor size (Figure 6I). As for healthy mice, both PEG‐ (Figure 6J) and TPP‐coated NPs (Figure 6K) mainly accumulated in the liver and spleen. One hour postinjection, TPP‐coated NPs were found in higher amounts compared to PEG‐coated ones, with lanthanum concentrations of 62 and 6%ID g^−1^ in the spleen, and 29 and 13%ID g^−1^ in the liver, respectively. For PEG‐coated NPs, the lanthanum concentration increased over time in liver and spleen, whereas it decreased in heart, lungs, and kidney. The highest tumor accumulation was measured 4 h after injection and reached 1.4%ID g^−1^. For TPP‐coated NPs, there was no significant change in the lanthanum accumulation over time. The highest tumor accumulation was also obtained 4 h postinjection and reached 0.6%ID g^−1^.

## 3 Discussion

2D cell culture models were used to investigate uptake mechanisms of LaF_3_:Ce NPs. Both XRF microscopy and TEM imaging showed their accumulation in lysosomes. However, the coating had a strong influence on their behavior: as TPP‐coated NPs aggregate more than PEG‐coated NPs, and are better internalized by tumor cells. Altogether, these results point to an absorption mechanism by endocytosis. ICP‐MS quantitatively confirmed qualitative TEM observations: TPP‐coated NPs are absorbed more efficiently than PEG‐coated NPs in both cell lines. Internalization kinetics were also affected by the coating, with TPP‐coated NPs accumulating more rapidly in cells. These findings could be explained by the larger hydrodynamic diameter and higher absolute charge of TPP‐coated NPs compared to PEG‐coated NPs. These differences in efficacy and speed of internalization may explain several observations. First, it can justify why a similar concentration of NPs in culture medium leads to a higher toxicity with TPP‐coated NPs compared to PEG‐coated NPs. This also correlates well with the higher DEF observed in clonogenic assays when accumulation of NPs is greatest, after 24 h incubation with 5 mg mL^−1^ NPs, in agreement with the literature.^[^ ^45^, ^47^ ^]^ The only incubation condition for which no RDE was observed is associated with the lowest lanthanum uptake (MIA PaCa‐2 cells incubated with PEG‐coated NPs). 3D models used to evaluate the ability of NPs to penetrate tumor spheroids provided similar trends to those observed in 2D models. XRF measurements demonstrated that TPP‐coated NPs accumulated in higher concentration in spheroids than PEG‐coated NPs. For both coatings, a fluorescence signal from the core of the spheroids was detected, demonstrating the diffusion of NPs in spheroids. Measurements of lanthanum and cerium concentrations using XRF microtomography and ICP‐MS showed that TPP‐coated NPs internalized more efficiently in spheroids than PEG‐coated NPs. However, as previously mentioned, concentrations measured by ICP‐MS for TPP‐coated NPs may have been overestimated. Treatment efficacy was evaluated at the highest nontoxic concentration determined beforehand: 24 h with 1 mg mL^−1^ NPs. Live/dead assays demonstrated a significant radiation dose‐enhancement for both cell lines, with a decrease in spheroid viability, more pronounced with TPP‐ compared to PEG‐coated NPs. In addition, while spheroid necrosis was induced by both types of NPs upon X‐ray irradiation in PANC‐1 spheroids, PEG‐coated NPs were more efficient in MIA PaCa‐2 ones. Regarding the effect on spheroid size, X‐ray irradiated PEG‐coated NPs only reduced the size of PANC‐1 spheroids. The enhanced therapeutic efficacy observed in PANC‐1 spheroids can be related to the greater accumulation of NPs. Indeed, a strong correlation was observed between the experimental DEF and the lanthanum concentration measured by ICP‐MS, both in 2D and 3D models, as shown Figure 5. These results confirmed the ability of LaF_3_:Ce NPs to increase the radiation dose when sufficiently accumulated in cancer cells and highlighted the role of the coating in fulfilling this condition. The therapeutic effect obtained with nanoscintillators is probably due to multiple reactions. The physical RDE effect is followed by chemical reactions leading to the production of ROS in the culture medium, and biological reactions leading to tumor cell death and DNA damage.^[^ ^48^ ^]^ Indeed, in the literature, in vitro and in vivo dose‐enhancement results have exceeded predictions made by Monte Carlo simulations, highlighting the strong contribution of chemical and biological steps. In this study, the coating was shown to strongly impact cell internalization in vitro and consequently the ability of the NPs to enhance the radiation dose in 2D and 3D models of pancreatic tumors. However, this parameter is also critical to ensure proper in vivo biocompatibility of NPs. According to plasma biochemistry, intravenous injection of LaF_3_:Ce NPs (200 mg kg^−1^) was well tolerated in mice with both coatings. It did not affect renal nor muscular functions, metabolism or nutritional status, and induce a mild hepatic toxicity after 14 days. Once injected, PEG‐ (≈45 nm) and TPP‐coated NPs (≈257 nm) mainly accumulated in the liver and spleen, in line with the literature. While NPs smaller than 10 nm undergo renal clearance, NPs larger than a few tens of nanometers undergo hepatic clearance.^[^ ^49^ ^]^ In accordance with their larger size, TPP‐coated NPs accumulated more in the spleen compared to PEG‐coated NPs. All these observations are coherent with previous in vivo studies of nanoscintillators in rodents that showed a preferential accumulation in the mononuclear phagocytic system.^[^ ^30^, ^38^, ^50^, ^51^, ^52^, ^53^, ^54^, ^55^ ^]^ Unlike TPP‐coated NPs, PEG‐coated NPs were progressively eliminated: lanthanum concentration measured in all organs was lower 14 days after injection compared to 24 h. Thus, the safety profile of TPP‐coated NPs is less appropriate than that of PEG‐coated NPs for the treatment of pancreatic cancer by intravenous injection, due to their high and irreversible nonspecific accumulation in liver and spleen. Because of their proximity to the pancreas, it would be difficult to specifically deliver X‐rays to a pancreatic tumor without irradiating the liver and spleen that would thus get damaged. These results point out that despite more promising in vitro properties, TPP‐coated NPs are less appropriate for in vivo applications. PEG‐coated NPs circulated twice as long as TPP‐coated NPs in the bloodstream and were associated with higher lanthanum concentration in the blood, reaching 25%ID g^−1^ 5 min after injection compared to less than 1%ID g^−1^ for TPP‐coated NPs. A longer blood circulation time is generally associated with greater tumor accumulation through the EPR effect.^[^ ^38^, ^54^ ^]^ This result was confirmed when assessing biodistribution in nude mice bearing orthotopic pancreatic tumors. Lanthanum accumulation in the tumor 1 and 4 h after injection was 2.3 times higher for PEG‐ than TPP‐coated NPs, and 10 times higher 24 h after injection. For other organs, a decrease in heart, lungs, and kidney for PEG‐coated NPs indicated an efficient elimination from nonspecific organs, which is consistent with the first biodistribution study performed in healthy mice. For liver and spleen, a progressive accumulation was observed, which may be explained by a prolonged circulation time in the blood. On the contrary, no significant evolution was observed for TPP‐coated NPs, in agreement with a much shorter circulation time. Because of their aggregation, TPP‐coated NPs may have been rapidly trapped in the liver and spleen, where more than 90%ID g^−1^ of lanthanum was accumulated 1 h after injection.

Thus, PEG‐coated NPs are more biocompatible with less nonspecific accumulation in healthy organs and a better elimination over time. They also exhibit a longer blood circulation time resulting in a better tumor accumulation, probably through EPR effect, making them more suitable for in vivo applications. These properties are in good agreement with the so‐called stealth effect of PEGylated nanomaterials.^[^ ^40^ ^]^ For future development, it is, however, important to remember that anti‐PEG immunity is a real challenge,^[^ ^56^ ^]^ as illustrated in a recent study that found that 56–72% of human serum samples presented anti‐PEG antibodies.^[^ ^57^ ^]^ For patients presenting anti‐PEG immunity, the circulation time of the nanotherapeutics will be strongly impaired,^[^ ^58^, ^59^ ^]^ which may jeopardize the efficacy of the treatment. Alternative approaches based, for example, on poly(ethyl ethylene phosphate) are being investigated to guarantee stealth effect while avoiding immunogenicity.^[^ ^60^ ^]^

Importantly, LaF_3_:Ce NPs are only toxic under X‐ray irradiation, as demonstrated in vitro where intracellular concentrations of lanthanum reached 1.4 × 10^−5^ and 1.5 × 10^−6^ μg cell^−1^ in PANC‐1 and MIA PaCa‐2 cells, respectively, without toxicity. Because RT can be spatially targeted, nontoxic accumulation in distant healthy organs can be tolerated. However, as previously discussed, accumulation in the liver and spleen following injection is problematic for pancreatic tumors because of the close proximity of these two organs with the pancreas. However, this may be less of a problem for tumors located further away from the spleen and liver, to which irradiation could be specifically delivered. Additionally, future studies will focus on improving NP biodistribution in order to improve intravenous administration and facilitate future clinical translation. However, if this goal is not achieved, intratumor delivery of LaF_3_:Ce NPs could be considered, using endoscopic delivery, as recently performed in human to deliver HfO_2_ NPs to a pancreatic tumor.^[^ ^61^ ^]^

LaF_3_:Ce NPs have shown promising characteristics for cancer treatment due to their low intrinsic toxicity, significant radiation dose‐enhancement, and good biocompatibility profile. Therefore, they remain promising candidates for other poor‐prognosis cancers, such as glioblastoma or ovarian cancer, which would benefit from the radiotherapeutic properties of nanoscintillators. However, for these two tumor types, different considerations may guide coating optimization, such as the ability to cross the blood–brain barrier and the capacity to specifically accumulate in the tumor versus surrounding tissue.

## 4 Conclusion

Because they enhance radiation dose in several tumor models, LaF_3_:Ce NPs are promising for X‐ray activated therapies. This work confirms that their coating strongly impacts both in vitro and in vivo properties, by affecting internalization and therapeutic efficacy of the NPs. Additionally, it shows that despite lower in vitro treatment efficacy, PEG‐coated NPs are better suited for therapeutic applications due to their improved biocompatibility properties. However, based on the biodistribution results, these NPs appear to not be relevant for intravenous injection for pancreatic cancer, but could hold promise for several other poor‐prognosis cancers, such as glioblastoma or ovarian cancer and/or for alternative injection routes, including a direct injection in the pancreatic tumor. The question of the coating is also to consider in regard with future perspectives of nanoscintillators for X‐ray‐induced photodynamic therapy or UV‐radioluminescence‐induced DNA damage that may require different intracellular distribution. Indeed, although intracellular localization of nanoscintillators may increase the efficacy of radiation dose‐enhancement, which relies on electrons that can travel up to a few tens of micrometers, it may not be necessary. However, the intracellular localization of nanoscintillators may play a more important role for X‐ray‐induced photodynamic therapy as the mean free path of the ROS produced, singlet oxygen for instance, is only of the order of a few nanometers. The proximity of the NPs to the intracellular target may therefore be more crucial.

## 5 Experimental Section

5.1

5.1.1

##### Synthesis of LaF_3_:Ce NPs: Materials

All reagents (lanthanide chlorides, purity: 99.99%, Jiayuan Advanced Materials Co., Ltd., China; hydrofluoric acid, ≃50 wt% in H_2_O, Alfa Aesar, France), solvents (2‐pyrrolidinone, methyl alcohol, Sigma–Aldrich Chemie, France), and functionalizing molecules (TPP, Sigma–Aldrich Chemie, France and polyethylene oxide (PEO) phosphonic acid, *M*_W_ = 1000–1250 g mol^−1^, Specific Polymers, France) of reagent‐grade quality were purchased and used without further purification.

##### Synthesis of LaF_3_:Ce NPs: Preparation of Highly Water‐Dispersible Cerium‐Doped Lanthanum Fluoride NPs

10.36 g (0.0279 mol) of lanthanum (III) chloride heptahydrate (LaCl_3_.7H_2_O) and 1.15 g (0.0031 mol.) of cerium (III) chloride heptahydrate (CeCl_3_.7H_2_O) were dissolved in 30 mL of methyl alcohol (Solution A). In parallel, 2.43 mL (0.0698 mol) of hydrofluoric acid were mixed with 124 mL of 2‐pyrrolidinone (Solution B). Solution A was quickly added to solution B, under magnetic stirrer. After 5 min stirring, a clear, colorless solution was obtained. The reaction medium was transferred into a Teflon‐lined stainless‐steel autoclave (Berghof, Germany, inner volume: ≈250 mL). The pressure vessel was sealed, heated, and maintained for 1 h at 170 °C under stirring. After cooling, the resulting reaction crude was poured into ≈160 mL acetone. La_0.9_Ce_0.1_F_3_ NPs precipitated immediately and completely as white flakes. These flakes were then isolated by centrifugation (8000 rpm, 20 min). The slightly yellow supernatant was carefully removed and the light‐brown centrifuged pellet was dispersed in 7 mL deionized (DI) water using an ultrasonic bath. The NPs were precipitated again in 50 mL acetone. This process was repeated twice. The final light‐brown pellet was dispersed in 6 mL DI water to obtain a transparent, slightly brown colloidal solution with a solid content of around 40% w/w.

##### Synthesis of LaF_3_:Ce NPs: La_0.9_Ce_0.1_F_3_ NP Functionalization

LaF_3_:Ce NPs were functionalized to improve their dispersion in PBS, using molecules that present phosphonate or phosphate groups, which have an affinity for the NPs surface, enriched in lanthanide ions during synthesis (excess of Ln^3+^ ions compared to fluorine ions).

##### Synthesis of LaF_3_:Ce NPs: PEG Grafting

An aqueous colloidal solution at 10% weight was prepared and introduced under vigorous stirring into an aqueous solution containing PEO phosphonic acid (PEG, 0.5 mol L^−1^). The Ln^3+^/P molar ratio was equal to 3. The resulting perfectly transparent and slightly brown solution was heated for 1 h at 80 °C. After cooling, the solution was dialyzed. The purified NPs were recovered by lyophilization and dispersed in PBS with a NP concentration of 35 mg mL^−1^.

##### Synthesis of LaF_3_:Ce NPs: TPP Grafting

An aqueous colloidal solution at 10% by weight was prepared. A 0.4 mol L^−1^ TPP aqueous solution was prepared and added with vigorous stirring to the colloidal solution. The Ln^3+^/P molar ratio was equal to 1.6. The resulting brownish and opalescent solution was stirred at room temperature for 4 h. The modified NPs were purified by ethanol precipitation, centrifugation, and dispersion in DI water. These steps were repeated 3 times before dispersing the final pellet in PBS with a final NP concentration of 35 mg mL^−1^.

##### NP Characterization

XRD patterns were recorded using a Malvern Panalytical Empyrean X‐ray diffractometer (Cu K*α* radiation at 0.154184 nm) equipped with a Ni filter and a PIXcel3D detector. Data were collected in the 2*θ* range of 10°–70°, with a scan speed of 0.5° min^−1^ and a 0.02° step width.

TEM analyses were carried out using a JEOL JEM 2100F TEM operating at 200 kV and equipped with a Gatan Ultrascan 1000 CCD camera and an Oxford X‐Max 80 mm^2^ EDS. Four different modes were used to characterize sample morphology, composition, and structure: TEM and high‐resolution TEM imaging, selected‐area electron diffraction, and scanning transmission electron microscopy–high‐angle annular dark‐field imaging coupled with EDS. Samples were prepared by depositing the NPs on 300 mesh copper grids coated with an ultrathin carbon film.

FTIR analysis was performed using a PerkinElmer Spectrum 100 FTIR spectrophotometer equipped with an ATR sample chamber.

Thermogravimetric analyses were performed on a Setaram LABSYS1600 system.

NMR experiments were performed on a Bruker Avance III 400 MHz spectrometer equipped with a Prodigy Cryoprobe. ^1^H, ^13^C, and ^31^P were acquired in deuterated water. Chemical shifts are given in ppm, using residual ethanol as a secondary reference. PEG and TPP molecules in deuterated water were also analyzed for comparison.

DLS measurements were performed using a Zetasizer Nano ZS (Malvern Panalytical). NPs solutions were diluted to reach 0.35 mg mL^−1^ for the measurements in PBS and culture medium and 0.20 mg mL^−1^ in mouse plasma. The parameters chosen for the analysis are described in **Table** **3**.

**Table 3:** Parameters used for DLS analysis

	Viscosity [cP]	Refractive index	Dielectric constant
Culture medium + 10% FBS	0.94	1.345	80
Mouse plasma	1.25	1.351	100

Zeta potentials (*ζ*) were measured with a Zetasizer Nano ZS (Malvern Panalytical) at a concentration of 0.35 mg mL^−1^ in PBS (pH = 7).

##### Cell Culture: Materials

PANC‐1 and MIA PaCa‐2 human pancreatic cancer cells were obtained from the American Type Culture Collection. The two cell lines were cultured in Dulbecco's Modified Eagle's Medium (DMEM, Gibco, 31966‐021) supplemented with fetal bovine serum (FBS: 10%, Dominique Dutscher, 500105M1M) and penicillin/streptomycin (1%, Gibco, 15140‐122), hereafter referred as complete medium. Cells were passaged twice a week at a 1:8–1:10 ratio, with passage number under 30, by using trypsin (trypsin‐EDTA 0.5% (10×), Gibco, 15400‐054). Cells were kept in a culture incubator maintained at 37 °C, 5% CO_2_.

The concentrations of LaF_3_:Ce NPs given hereafter correspond to the final concentration in NPs in complete medium. For control conditions, the medium was replaced or supplemented with fresh complete medium without NPs.

##### Cell Culture: Spheroid Model

The spheroids were grown in suspension in round bottom ultralow attachment plates (96‐well, Corning). 5000 cells were seeded per well in 100 μL medium in each well. The outer wells of the plate were filled with PBS to prevent evaporation. Cultures were maintained for 24 h in the incubator to allow spheroid formation. NPs incubation was performed by replacing half of the culture medium by a 2× solution of LaF_3_:Ce NPs.

##### XRF Microscopy and Cryo‐Optical Fluorescence Microscopy

PANC‐1 cells were seeded as adherent monolayers on silicon nitride membranes (Silson) deposited in the bottom of 4‐well plates for 24 h, and were incubated for another 24 h with LaF_3_:Ce NPs at 0.1 mg mL^−1^. After rinsing with PBS, the cells were incubated for 35 min at culture conditions with lysotracker green (50 nm, Invitrogen L7526) and Hoechst 33 342 (2 drops per milliliter according to the manufacturer's instruction, NucBlue, Invitrogen R37605). Cells were quickly rinsed with ammonium acetate buffer (150 mm), cryofixed (Leica, EM GP) and stored in liquid nitrogen until imaging. The vitrified cells were imaged at −185 °C using a widefield cryofluorescence light microscope (Leica, Cryo CLEM), with a THUNDER Imager system equipped with a ceramic‐tipped lens (NA = 0.9, 50×) using the brightfield and band pass filter cubes of green fluorescent protein (*λ*_em_ = 525 nm) and 4′,6‐diamidino‐2‐phénylindole (*λ*_em_ = 477 nm). The distribution of physiological elements (e.g., Zn, P) and lanthanum was measured by XRF microscopy at the ID16A beamline of the European Synchrotron Radiation Facility (ESRF, Grenoble, France). More details about the imaging parameters and analysis are provided in S2, Supporting Information.

##### XRF Microtomography

Spheroids of PANC‐1 cells were grown in suspension for 24 h as previously described, and incubated for another 24 h with LaF_3_:Ce NPs at 0.1 mg mL^−1^. Spheroids were rinsed with PBS, fixed in PFA, and glued on 200 μm diameter quartz capillaries, as previously described.^[^ ^30^ ^]^ XRF microtomography imaging was performed at the P06 beamline of the storage ring PETRA III (Deutsches Elektronen‐Synchrotron DESY, Hamburg) (Figure S11, Supporting Information). The samples were imaged after excitation with a monochromatic beam (14 keV), which was focused down to a size of about 450 nm × 300 nm (h × v) using Kirkpatrick–Baez mirrors. Images were acquired with a resolution of 5 μm/pixel, an acquisition time of 4 ms/pixel, and using 120 projections over 0°–360.5° (acquisition time/spheroid ≈7 h). For each scan point of the measurements, the XRF spectra were fitted using the nonlinear least squares method implemented in the PyMca software.^[^ ^62^ ^]^ The fitted XRF data were then sorted into image grids and quantified using calibration foils, either with known area density or in combination with parameters provided by the xraylib library,^[^ ^63^ ^]^ according to the elements. Tomographic reconstruction of the quantified XRF data was performed using a maximum‐likelihood expectation‐maximization algorithm.^[^ ^64^ ^]^ The final data provided the concentration for various elements including zinc (Zn), naturally present in cell nuclei and lanthanum (La).

##### TEM Imaging on Cells

PANC‐1 and MIA PaCa‐2 cells were grown as adherent monolayers on 2‐well Lab‐Tek (Thermo Scientific, 177380) for 2 days. Cells were incubated for 24 h with 0.1 mg mL^−1^ NPs. After incubation, the cells were washed with DMEM and fixed for 30 min in a solution containing 2% PFA (R1026) and 0.2% glutaraldehyde (GA, R1020) in DMEM with gentle shaking. After successive fixation, rinsing and staining steps as previously described,^[^ ^65^ ^]^ cells were dehydrated in graded ethanol series, and embedded in Epon resin (Embed 812, Electron Microscopy Sciences, 14120). Ultrathin sections of 70 nm were cut on an ultramicrotome (Leica, UC7), collected on formvar‐carbon‐coated copper 100 mesh grids (Formvar) and imaged with a Tecnai G2 Spirit BioTwin, with magnifications from 690× to 9300×.

##### MTS Assay

7500 cells/well were seeded in 96‐well plates. After 24 h, NPs were added to the cultures for 24 h, with concentrations ranging from 0.01 to 20 mg mL^−1^. Untreated controls were prepared with complete medium and total killing controls were prepared by adding dimethyl sulfoxide (10%, Sigma–Aldrich, D4540). Cells were rinsed with PBS, and 100 μL of viability solution (CellTiter 96 Aqueous One Solution Cell Proliferation Assay, Promega, G3581) was added to each well already containing 100 μL of medium. After 2 h incubation, the absorbance was measured at 490 nm with a CLARIOstar (BMG Labtech) plate reader.

##### X‐ray Irradiation of the Cell Cultures

X‐rays were delivered by a CIX2 irradiator (Xstrahl) through a 3 mm aluminum filter, with the following parameters: voltage = 195 kV, current = 10 mA, and focal source distance = 40 cm. The cell culture plates were placed at the center of the irradiation field to ensure a uniform radiation dose. The dose rate was measured using a PTW ionization chamber (TN30010‐1) and a PTW UNIDOS E electrometer. The typical dose rate in water for these experiments was 1.8 ± 0.5 Gy min^−1^.

##### Clonogenic Assay

Cells were seeded in T25 flasks and maintained in the incubator for 3 days. They were incubated for 1 h with 2 mL of LaF_3_:Ce NPs at 5 mg mL^−1^ and irradiated as previously described. After irradiation, cells were rinsed 3 times with PBS and collected using trypsin. Viable cells were counted with an automated cell counter (NanoEnTek EVE) using trypan blue solution (0.4%, Invitrogen, T10282). 2 mL of complete medium was placed in each well of a 6‐well plate, and cells were seeded at various densities according to the irradiation dose received (Table S2, Supporting Information). Cells were maintained at 37 °C, 5% CO_2_ for 2 weeks to allow the colonies to grow, with medium renewal when necessary. Colonies were then rinsed with PBS and incubated for 30 min with a staining solution of 0.5% crystal violet (Amresco, 0528) and 6% GA (Sigma–Aldrich, G6257) diluted in water. Plates were rinsed with water until the bottom was clear. Pictures were taken and analyzed using a custom‐made ImageJ‐based software.

##### Live/dead Assay in 3D Models

A live/dead assay was used to measure spheroid viability using a double staining based on calcein AM (live cells) and propidium iodide (PI, necrotic cells), as previously described.^[^ ^46^, ^66^ ^]^

##### Live/Dead Assay in 3D Models: Toxicity in Spheroids

Spheroids were grown as previously described and incubated for 24 h with increasing concentrations of LaF_3_:Ce NPs ranging from 0.01 to 20 mg mL^−1^ in culture medium. Control and NP‐incubated spheroids were carefully rinsed twice by gently replacing 150 μL of the existing medium by the same volume of complete medium. A total killing group with 100% necrotic cells was prepared by fixing the spheroids for 2 min in formalin solution (10% neutral buffered, Sigma–Aldrich, HT5012). Spheroids were then rinsed with PBS and incubated with a 0.5% Triton solution (Triton X‐100, Bio‐Rad, 161‐0407) in PBS for 30 min to permeabilize the membranes. Spheroids were rinsed twice with a 0.1 mol L^−1^ glycine solution (Euromedex, 26‐128‐6405C) and maintained in PBS. After these steps, half of the medium was removed and replaced with the same volume of a live/dead staining solution (4 μmol L^−1^ calcein green AM, Invitrogen, C34852 and 6 μmol L^−1^ PI, Sigma–Aldrich, P4864) prepared in PBS. The plates were maintained in the incubator for 1.5 h in the dark before being imaged using a confocal microscope (Zeiss, LSM510 Confocor II Combi) with a 5× objective (Plan Neofluar, NA = 0.15). The live (calcein) and dead (PI) signals were recorded at *λ*_exc_ = 488 nm/*λ*_em_ = 500–540 nm and *λ*_exc_ = 543 nm/*λ*_em_ = 600–670 nm, respectively. Image processing was performed using the custom‐developed CALYPSO MATLAB Code.^[^ ^66^ ^]^

##### Live/Dead Assay in 3D Models: Therapeutic Efficacy in Spheroids

Spheroids were grown as previously described and incubated for 24 h with LaF_3_:Ce NPs at 1 mg mL^−1^. Then, they were irradiated at 4, 8, or 12 Gy and rinsed twice by gently replacing 150 μL of the medium by the same volume of complete medium. Spheroids were carefully transferred using wide‐orifice tips to new wells containing 200 μL of complete medium. Plates were maintained in culture conditions for 5 days. At that time, 145 μL of existing medium was removed to leave 50 μL of complete medium in each well. A 50 μL volume of live/dead staining solution prepared as previously described was added to each well. After 1.5 h incubation, spheroids were imaged as previously described.

##### ICP‐MS Measurements on Cell Samples

Lanthanum and cerium concentrations were determined using quadrupole ICP‐MS (Perkin Elmer NexION 2000, Waltham, MA, USA). Adherent cells were seeded and grown following the clonogenic assay protocol. After NP incubation and rinsing steps previously described, cells were collected using trypsin, centrifuged, and counted. The cell pellet was resuspended in 500 μL PBS. Spheroids were seeded following the live/dead assay protocol. After NPs incubation, spheroids were rinsed as previously described, collected using large‐orifice tips, and manually dissociated to obtain a homogeneous cell suspension. Cells were counted, the suspension was centrifuged, and the cell pellet was resuspended in 200 μL PBS. All samples were stored at −20 °C until analysis. Samples were mineralized under atmospheric pressure in nitric acid for 24 h at room temperature, followed by three phases of 8 h in an oven (50 °C) over 3 consecutive days. The mineralization was diluted to reach 1% concentration of nitric acid before analysis. Standard solutions were prepared in nitric acid 1% v/v. ^139^La and ^140^Ce were measured; ^103^Rh was used as an internal standard.

##### Animal Experiments

All animal studies were performed in accordance with European guidelines and under the approval of the local ethics committee and the French Ministry of High Education and Research under the reference Apafis #32222‐2022050416335097 v2. The animals were examined daily for general condition and behavior, and their weight was monitored 3 times a week. Humane endpoints were defined in advance to prevent, terminate, or relieve animal pain or distress. Intravenous injections (200 μL) were performed at ≈200 μL min^−1^ in the tail vein under isoflurane anesthesia (3%). Control groups received a PBS injection in the same conditions. Euthanasia was performed by cervical dislocation under 4% isoflurane anesthesia.

##### Animal Experiments: Toxicity

Six week old BALB/cJRj female mice (Janvier labs, France) received an intravenous injection of LaF_3_:Ce NPs (200 μL, 200 mg kg^−1^), *N* = 5 animals/condition. Approximately 200 μL of blood was collected 24 h and 14 days postinjection for plasma biochemistry analysis. Blood was centrifuged for 5 min at 10 000 rpm (20 °C) to isolate the plasma. Samples were then kept at −80 °C until analysis, which was performed using a M‐Scan II (Melet Schloesing Laboratories) and the VET‐16 reagent rotors that include markers of different physiological parameters. To measure NP biodistribution, mice were euthanized 24 h or 14 days after injection. Organs (heart, liver, lungs, left kidney, and spleen) were harvested, weighted, and stored at −80 °C. The organs were mineralized in 300 μL of concentrated HNO_3_ and the lanthanum and cerium concentrations were measured by ICP‐MS.

##### Animal Experiments: Pharmacokinetics

Six week old BALB/cJRj female mice (Janvier labs, France) received an intravenous injection of LaF_3_:Ce NPs (200 μL, 200 mg kg^−1^), *N* = 3 animals/condition. Approximately 20 μL of blood was collected at the tail end: 5, 15, 30 min; 1, 2, 4, 8, and 24 h postinjection. Blood samples were stored at −80 °C until ICP‐MS measurements.

##### Animal Experiments: Biodistribution

Six week old NMRI female nude mice (Janvier labs, France) were anesthetized (air/isoflurane 4% for induction, 2.5% during surgery) and buprenorphine (0.1 mg kg^−1^) was injected subcutaneously. The left flank was incised and the spleen gently pulled out, exposing the pancreas. One million of luciferase‐expressing PANC‐1 cells were resuspended in 50 μL Matrigel solution and orthotopically injected into the pancreas using a 29G syringe. After injection, the spleen and pancreas were repositioned and the different planes were sutured. Noninvasive bioluminescence imaging was performed 34 days after tumor cell injection (LUMINA III, PerkinElmer) to assess the presence of the tumor and randomize the animals in the different treatment groups (5 animals/group). All data are provided Figure S10, Supporting Information. Twenty‐four hours later, mice received an intravenous injection of LaF_3_:Ce NPs (200 μL, 200 mg kg^−1^). Mice were euthanized 30 min, 4, or 24 h after injection of NPs, and 30 min after PBS injection. Organs (heart, liver, lungs, left kidney, spleen, and pancreas) and tumors were harvested, weighted, and stored at −80 °C until ICP‐MS analysis for lanthanum and cerium quantification.

##### Statistical Analysis and Regression Fits

Statistical analyses were performed using GraphPad Prism version 9.2.0 (GraphPad Software, San Diego, California USA). All in vitro experiments were repeated independently at least twice. Results are presented as mean ± SEM (standard error of the mean). Student's *t*‐test (two‐tailed unpaired) was used to compare two experimental groups, especially PEG and TPP coatings for in vitro ICP‐MS analysis. One‐way analysis of variance (ANOVA) followed by Tukey post hoc test was used for multiple‐group comparison with one factor, in particular for the XRF microtomography experiments. Two‐way ANOVA followed by Tukey post hoc test was used for multiple‐group comparison with two factors, including clonogenic and live/dead assays, as well as plasma biochemistry analysis. The threshold for statistical significance was set at a *p*‐value below 0.05; (*) indicates *p* < 0.05, (**) indicates *p* < 0.01, and (***) indicates *p* < 0.001.

The nonlinear regression fits for toxicity experiments (MTS for 2D models and live/dead assays for 3D models) were calculated according to the [inhibitor] versus response model, with variable slope and four parameters. The half maximal inhibitory concentrations IC_50_ were calculated from the nonlinear regression fits. The nonlinear regression fits for clonogenic assays were calculated according to the linear quadratic cell death model. The linear regression fits for live/dead assays were calculated according to the simple linear regression model. The nonlinear regression fits for blood pharmacokinetics experiments were calculated according to the two‐phase decay model.

## Conflict of Interest

The authors declare no conflict of interest.

## Supporting information

Supplementary Material

## Data Availability

The data that support the findings of this study are available from the corresponding author upon reasonable request.
