# Supplementary material for: Nanoscintillator Coating: A Key Parameter That Strongly Impacts Internalization, Biocompatibility, and Therapeutic Efficacy in Pancreatic Cancer Models
Source: Small Sci. 2024 Mar 28;4(5):2400041. doi: 10.1002/smsc.202400041 (PMC11935242; doi:10.1002/smsc.202400041)
Supplement: Supplementary file 1 — Supplementary Material [file SMSC-4-2400041-s001.pdf]

Supporting Information

Nanoscintillator coating: a key parameter that strongly impacts internalization, biocompatibility and therapeutic efficacy in pancreatic cancer models

*Clémentine AUBRUN FULBERT *, Frédéric CHAPUT, Sarah STELSE-MASSON, Maxime HENRY, Benoit CHOVELON, Sylvain BOHIC, Dennis BRUECKNER, Jan GARREVOET, Christine MORISCOT, Benoit GALLET, Julien VOLLAIRE, Olivier NICOUD, Frédéric LEROUGE, Sandrine DENIS-QUANQUIN, Xavier JAURAND, Thibault JACQUET, Anthony NOMEZINE, Véronique JOSSERAND, Jean-Luc COLL, Jean-Luc RAVANAT, Hélène ELLEAUME [&] and Anne-Laure BULIN [&,*]*

SI. 1. ^{31}P Nuclear Magnetic Resonance (RMN)

All samples were prepared in deuterated water.

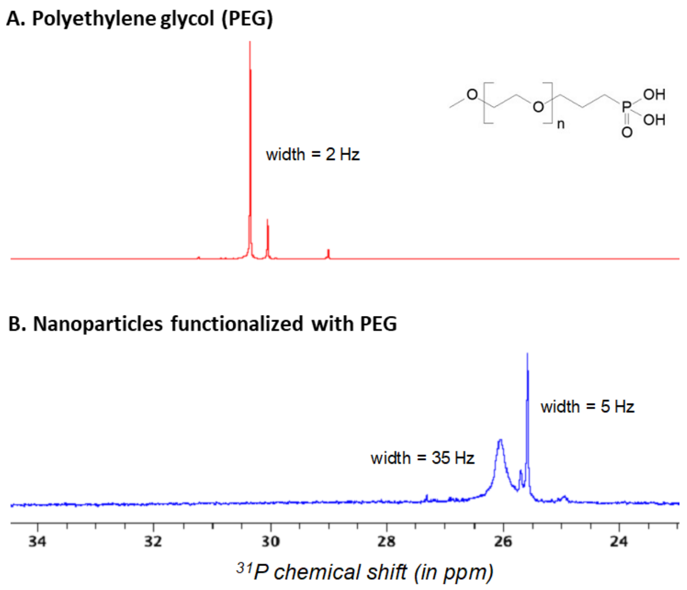

Figure S1. ^{31}P NMR spectra of polyethylene glycol (PEG, A) and LaF_3:Ce NPs functionalized with PEG (B).

PEG molecules exhibit one sharp signal at 30.5 ppm, whereas two signals are observed for functionalized NPs, at 26 and 25.5 ppm. The two signals are attributed to two binding modes of the phosphorus head on the surface, with probably one or two oxygens in interaction. The sharpness of the signals indicated non-covalent interactions.

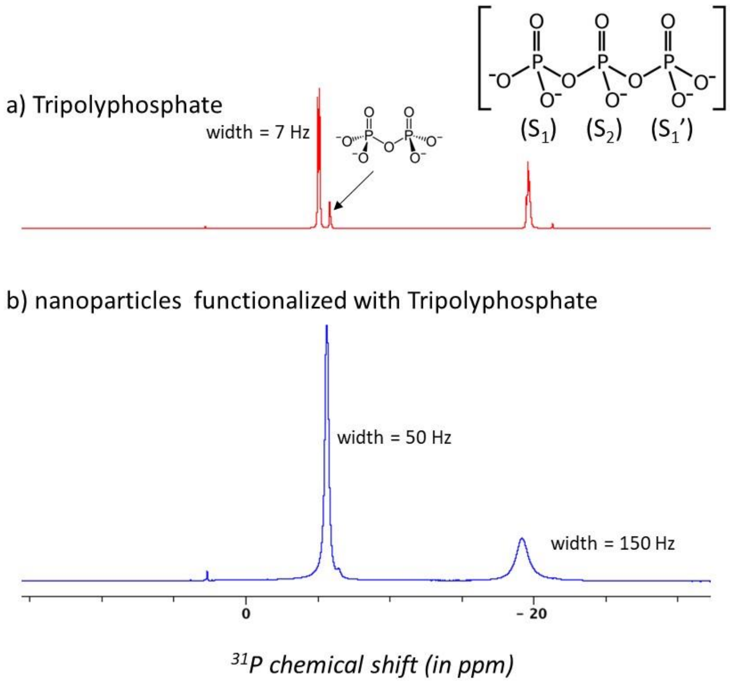

Figure S2. ^{31}P NMR spectra of tripolyphosphate (A) and LaF_3:Ce NPs functionalized with TPP (B).

TPP molecules exhibit two main signals at -5 and -19.5 ppm (presence of a weak signal at -5.8 ppm corresponding to pyrophosphate, here as an impurity), which were also observed for functionalized NPs with significant broadening, confirming the presence of the TPP on the nanoparticle surfaces. These signals can be assigned respectively to the terminal and middle phosphors of the TPP molecules. The NMR spectra suggest that the symmetry of the TPP molecule is preserved and no hydrolysis occurred during the reaction. Several coordination sites can be considered for the surface cations (S_1, S_2 or S_1'). Interaction with the nanoparticle surface takes place either through the S_2 site, leaving the P-O^- of the S_1 and S_1' sites free, or through the S_1 and S_1' sites, leaving the P-O^- of the S_2 site free. These coordination modes give the nanoparticles a negative surface charge, confirmed by zeta potential measurements in water at pH=7 (-45 mV). It

should also be noted that solution NMR measurements do not allow the observation of aggregated NPs, their signals being broadened to the point of being indistinguishable from the baseline.

SI. 2. X-ray fluorescence microscopy

ID16A beamline is under high-vacuum to enable measurements on frozen hydrated samples at -153°C. A monochromatic X-ray beam (17 keV) was focused down to 40 nm using a pair of Kirkpatrick-Baez mirrors. The emission of fluorescence was recorded by two custom multi-element Silicon Drift Detectors placed on both sides of the sample and facing each other at 90° from the incident X-ray beam. The resulting XRF spectra were fitted pixel by pixel (50 nm size, 50 ms dwell-time) using PyMca software [47] and the elemental areal mass concentration were calculated. Imaging a single cell took ≈ 4-5 hours. The correlative imaging between optical fluorescence microscopy and XRF elemental images was obtained using ec-CLEM plugin of Icy software.

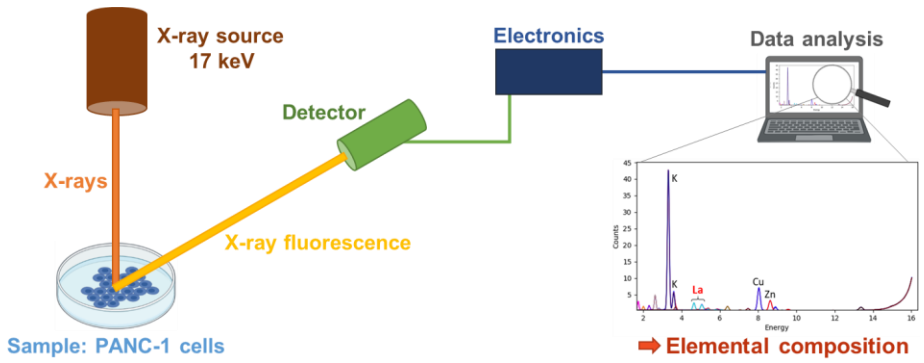

Figure S3. Schematic representation of X-ray fluorescence spectroscopy principle, as operated on ID16A, European Synchrotron Radiation Facility, Grenoble, France. Created with BioRender.com.

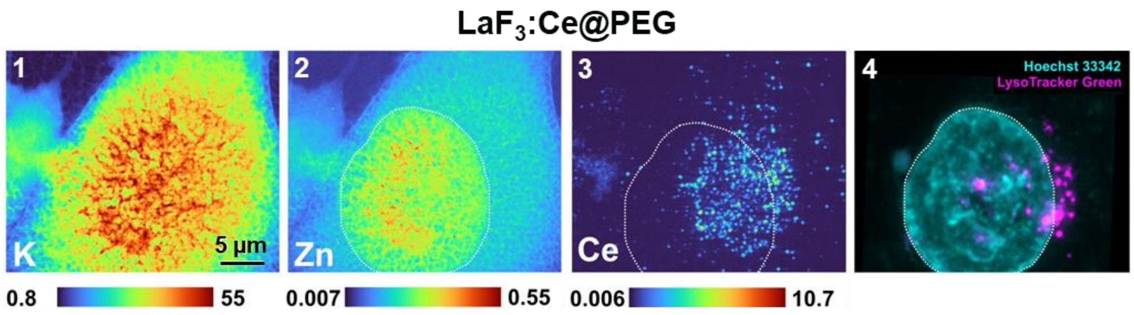

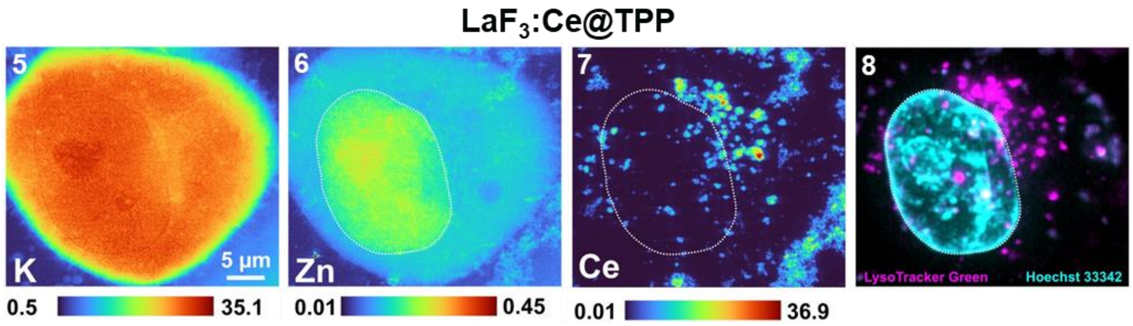

Figure S4. X-ray fluorescence spectroscopy images of PANC-1 cells incubated for 24 hours with LaF_3:Ce NPs at 0.1 mg.mL^{-1} (scale bar = 5 µm, ESRF, ID16), either with PEG (1-4) or TPP coating (5-8). Different elements are represented: 1, 5) potassium (cytoplasm), 2, 6) zinc (nucleus), 3, 7) cerium (NPs), 4, 8) Lysotracker Green (purple) and Hoechst 33342 (cyan).

SI. 3. ICP-MS results

		**LaF_3:Ce@PEG**		**LaF_3:Ce@TPP**	
**2D models**		24h, 1 mg.mL^{-1}	1 h, 5 mg.mL^{-1}	24 h, 1 mg.mL^{-1}	1 h, 5 mg.mL^{-1}
	**PANC-1**	1.81x10^{-5}	1.37x10^{-5}	6.40x10^{-5}	1.24x10^{-4}
	**MIA PaCa-2**	9.79x10^{-6}	1.47x10^{-6}	1.41x10^{-5}	9.11x10^{-5}
**3D models**		**LaF_3:Ce@PEG**		**LaF_3:Ce@TPP**	
		24 h, 1 mg.mL^{-1}		24 h, 1 mg.mL^{-1}	
	**PANC-1**	1.83x10^{-4}		1.30x10^{-2}	
	**MIA PaCa-2**	1.92x10^{-5}		2.33x10^{-3}	

Table S1. Intracellular lanthanum concentration (µg.cell^{-1}) measured in 2D and 3D models using ICP-MS

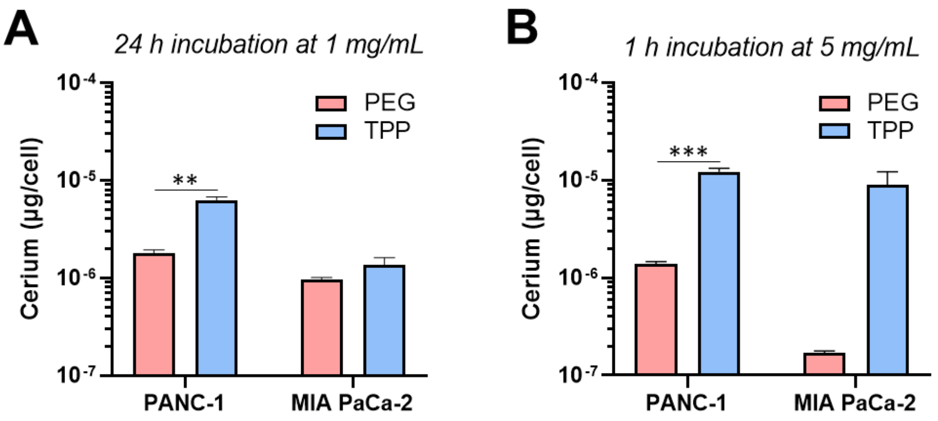

Figure S5. Concentrations of cerium measured by ICP-MS in PANC-1 and MIA PaCa-2 cells gown in 2D after incubations with LaF_3:Ce NPs: **A)** 24 hours at 1 $mg.ml^{-1}$, **B)** 1 hour at 5 $mg.mL^{-1}$.

SI. 4. Dose-enhancement factor (DEF)

SI.4.1 Experimental DEF calculation using clonogenic assays.

The survival plots of the control groups, normalized on the 0 Gy condition, were first fitted with the linear quadratic model (**Equation 1**), which provided the α and β parameters. The α (Gy^{-1}) parameter characterizes the initial slope of cell survival curves and low-dose efficacy, while β (Gy^{-2}) represents the increasing contribution of cumulative damage, assumed to be due to the interaction of two or more lesions. The α/β ratio represents the dose at which the two terms contribute equally to the effect [1], and are characteristic of each cell line.

$$\frac{S_{Controls}}{S_0} = e^{-(\alpha_c D + \beta_c D^2)} \qquad \textbf{Equation 1}$$

$\frac{S_{Controls}}{S_0}$ represents the normalized survival of the control cells, relative to 0 Gy; α_c (Gy^{-1}) and β_c (Gy^{-2}) are the characteristic parameters of each cell line, and D is the radiation dose (Gy).

We calculated the DEF assuming that the cells survival in presence of the nanoparticles was impaired only by a physical enhancement of the dose due to the presence of the NPs. The survival plots of the cells irradiated in presence of the NPs were fitted with **Equation 2** to extract the DEF, using the α and β parameters obtained from the control survival data of each cell line.

$$\frac{S_{nano}}{S_{nano,0}} = e^{-(\alpha_c.D.DEF + \beta_c.D^2.DEF^2)} \qquad \textbf{Equation 2}$$

$\frac{S_{nano}}{S_{nano,0}}$ is the normalized survival of the cells irradiated in presence of the NPs; α_c and β_c are the characteristic parameters of each cell line (described using equation 1) and D is the radiation dose (Gy).

SI.4.2 Experimental DEF from 3D live/dead assay.

To estimate the DEF from the experimental live/dead assay data performed on spheroids, the spheroid viability normalized on 0 Gy, plotted as a function of the X-ray dose were fitted with a linear model (**Equation 3**).

$$\frac{V}{V_0} = -\gamma \, . \, D \qquad \textbf{Equation 3}$$

Where $\frac{V}{V_0}$ is the viability of the cells normalized to the viability obtained at 0 Gy, γ (Gy^{-1}) the slope, and D is the radiation dose (Gy).

The DEF was obtained as the ratio of the slope of the viability plot obtained in presence of the NPs to the slope of the control curve for each cell line.

SI. 5. Therapeutic efficacy in 3D culture model

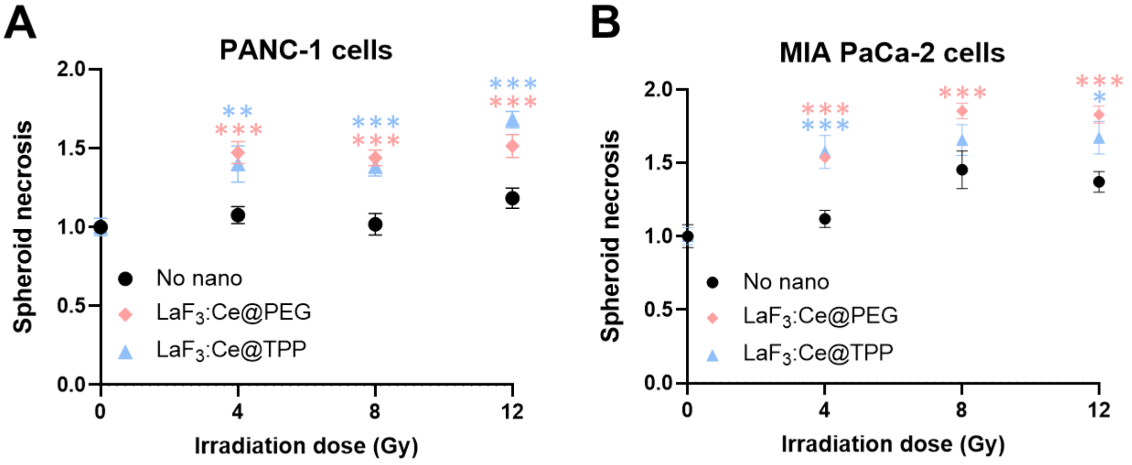

Figure S6. Spheroid necrosis plotted as a function of the irradiation dose for PANC-1 (A) and MIA PaCa-2 (B) cells. Data were normalized on the 0 Gy condition.

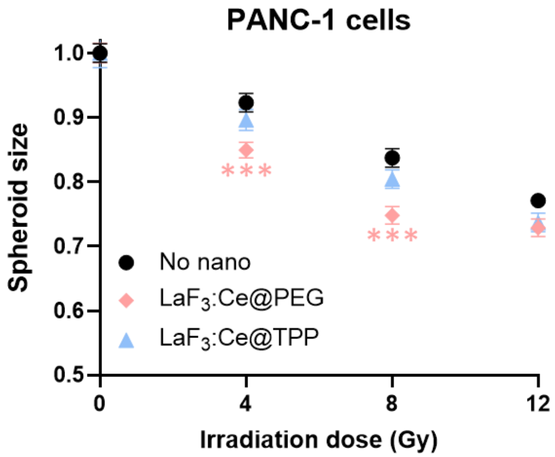

Figure S7. Size of the spheroids plotted as a function of the irradiation dose for PANC-1 spheroids. Data were normalized on the 0 Gy condition.

SI. 6. Animal monitoring

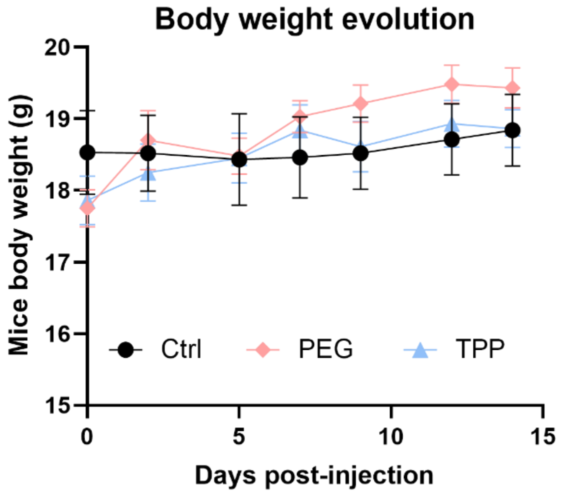

Figure S8. Body weight of BALB/c mice after intravenous injection of LaF_3:Ce NPs (200 $mg.kg^{-1}$, 200 µL), N = 5 mice/group.

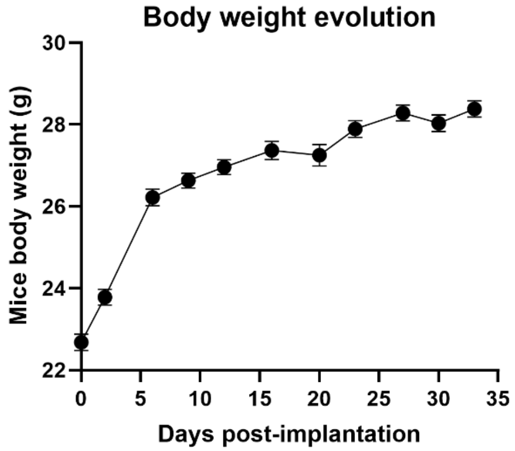

Figure S9. Body weight of NMRI nude mice after orthotopic implantation of pancreatic tumor cells, N = 5/group.

SI. 7. Randomization of the mice bearing orthotopically implanted pancreatic tumors for the biodistribution experiment

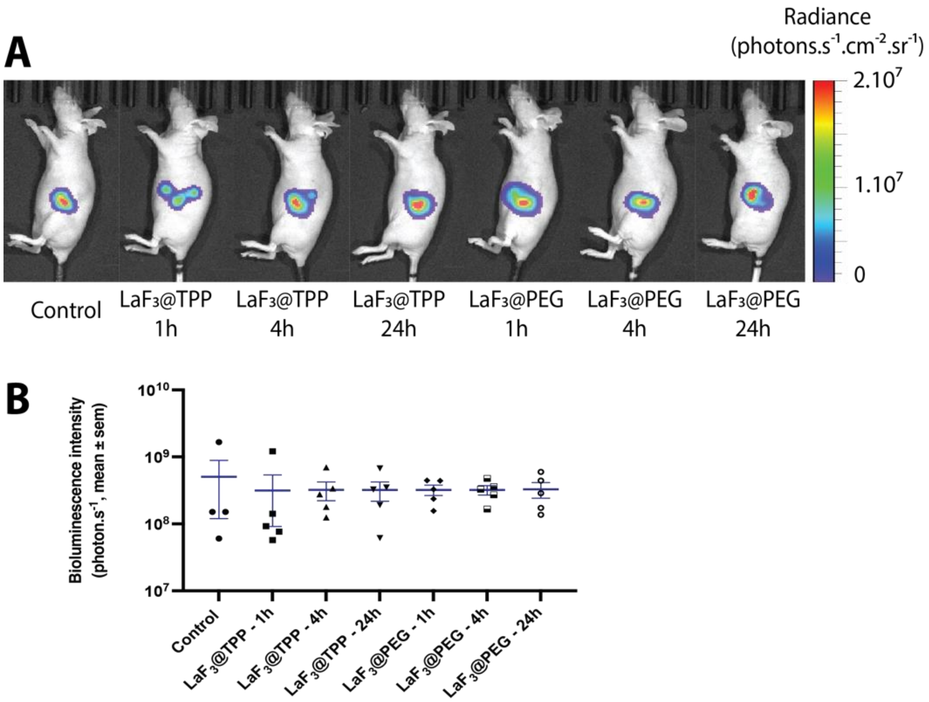

Figure S10. Animals baring orthotopically implanted pancreatic tumor were randomized using bioluminescence intensity measured 34 days post implantation (N = 5 mice/group). A) Representative images of mice bearing orthotopic pancreatic tumors 34 days post tumor implantation. **B)** The bioluminescence intensity was used to randomized the animals in each group.

SI. 8. X-ray fluorescence microtomography

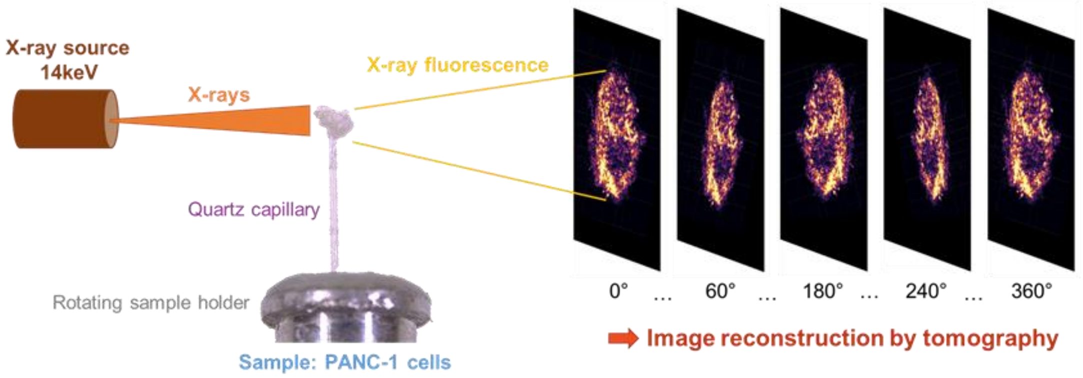

Figure S11. Schematic representation of X-ray fluorescence microtomography principle.

SI. 9. Experimental conditions for the clonogenic assays

	Dose (Gy)				
	0	1	2	3	4
PANC-1	200 / 250	225 / 275	450 / 550	800 / 1200	1800 / 2200
MIA PaCa-2	150 / 200	250 / 300	300 / 400	800 / 1200	1800 / 2200

Table S2: Number of cells seeded per well for various X-ray doses

[1] N. A. P. Franken, A. L. Oei, H. P. Kok, H. M. Rodermond, P. Sminia, J. Crezee, L. J. A. Stalpers, G. W. Barendsen, *Int. J. Oncol.* **2013**, *42*, 1501.
